# Biogeography of the Relationship between the Child Gut Microbiome and Innate Immune System

**DOI:** 10.1128/mBio.03079-20

**Published:** 2021-01-12

**Authors:** Nelly Amenyogbe, Pedro Dimitriu, Kinga K. Smolen, Eric M. Brown, Casey P. Shannon, Scott J. Tebbutt, Phillip J. Cooper, Arnaud Marchant, Tessa Goetghebuer, Monika Esser, Brett B. Finlay, Tobias R. Kollmann, William W. Mohn

**Affiliations:** a Department of Experimental Medicine, University of British Columbia, Vancouver, Canada; b Telethon Kids Institute, Perth, Australia; c Department of Microbiology and Immunology, Life Sciences Institute, University of British Columbia, Vancouver, Canada; d Michael Smith Laboratories, University of British Columbia, Vancouver, Canada; e PROOF Centre of Excellence, St. Pauls’s Hospital, University of British Columbia, Vancouver, Canada; f Centre for Lung Innovation, Department of Medicine, Division of Respiratory Medicine, University of British Columbia, Vancouver, Canada; g Facultad de Ciencias Medicas, de la Salud y la Vida, Universidad Internacional del Ecuador, Quito, Ecuador; h Institute of Infection and Immunity, St. George’s University of London, London, United Kingdom; i Institut d’Immunologie Médicale, Université Libre de Bruxelles, Charleroi, Belgium; j Département de Pédiatrie, Centre Hospitalier Universitaire St Pierre, Université Libre de Bruxelles, Brussels, Belgium; k Immunology Unit, Division of Medical Microbiology, Department of Pathology, NHLS and Stellenbosch University, Matieland, Stellenbosch, South Africa; l Department of Pediatrics, Division of Infectious Diseases, University of British Columbia, Vancouver, Canada; Yale School of Public Health

**Keywords:** biogeography, gut microbiome, innate immunity

## Abstract

The gut microbiome is a well-recognized modulator of host immunity, and its compositions differ between geographically separated human populations. Systemic innate immune responses to microbial derivatives also differ between geographically distinct human populations. However, the potential role of the microbiome in mediating geographically varied immune responses is unexplored. We here applied 16S amplicon sequencing to profile the stool microbiome and, in parallel, measured whole-blood innate immune cytokine responses to several pattern recognition receptor (PRR) agonists among 2-year-old children across biogeographically diverse settings. Microbiomes differed mainly between high- and low-resource environments and were not strongly associated with other demographic factors. We found strong correlations between responses to Toll-like receptor 2 (TLR2) and relative abundances of *Bacteroides* and *Prevotella* populations, shared among Canadian and Ecuadorean children. Additional correlations between responses to TLR2 and bacterial populations were specific to individual geographic cohorts. As a proof of concept, we gavaged germfree mice with human donor stools and found murine splenocyte responses to TLR stimulation were consistent with responses of the corresponding human donor populations. This study identified differences in immune responses correlating to gut microbiomes across biogeographically diverse settings and evaluated biological plausibility using a mouse model. This insight paves the way to guide optimization of population-specific interventions aimed to improve child health outcomes.

## Abstract (precis)

Both the gut microbiome and innate immunity are known to differ across biogeographically diverse human populations. The gut microbiome has been shown to directly influence systemic immunity in animal models.

## INTRODUCTION

The gut microbiome is a well-recognized modulator of host systemic immunity throughout life (1). Its compositions differ between geographically separated human populations (2–4). Systemic innate immune responses to microbes are largely driven by the stimulation of pattern recognition receptors (PRR), i.e., microbially derived agonists, triggering production of a range of cytokines. These immune responses have also been shown to differ between geographically distinct populations (5, 6). However, the specific role of the microbiome composition and its function in mediating these differing immune responses across geographical regions remain unknown. Given the ability of the host microbiome to modulate systemic innate immunity and the known geographical differences between both gut microbiomes and innate immune phenotypes, what is missing is a mechanistic understanding of how the distinct microbiomes likely contribute to immune differences. Robust correlations from human studies are therefore needed to inform mechanistic work using animal models.

Previous studies have found associations between systemic immunity and host microbiome within single cohorts, finding that relative abundance of microbial taxa or their genes could be correlated with select cytokine responses to Toll-like receptor (TLR) stimulation (7, 8). These studies relied on univariate statistics of relative abundance data to find a small subset of microbiome-immune correlations. Components of human microbiomes have also been shown to modulate immune phenotypes *in vitro* (9). However, univariate statistical methods may often lead to spurious results, as the independence assumption between predictor variables is not met. Furthermore, by considering only one-to-one associations, univariate approaches test each operational taxonomic unit (OTU) individually and disregard interactions or correlations among OTUs, providing limited insight into the system (10).

Direct comparisons of innate immune responses across different locations are hampered by the need to standardize and control all aspects of immune assessment to avoid technical artifacts. We previously applied a rigorously standardized approach to quantify cytokine responses to a panel of TLR agonists among 2-year-old children recruited in Brussels (Belgium), Cape Town (South Africa), Quininde (Ecuador), and Vancouver (Canada) (6). These four biogeographically distinct settings differ in many ways that can potentially modulate both systemic immunity and the gut microbiome, with resource availability, ancestry of the human populations, diet, climate, vaccination schedules, and cultural practices being examples. Hence, these sites were chosen to test the hypothesis that systemic innate immunity differs among diverse child populations. Recently, we found that differences in gut microbiomes and immune phenotypes between HIV-exposed and healthy HIV-unexposed children were specific to each cohort (11). Here, we extended this work by integrating the stool microbiomes of healthy children measured via sequencing of the 16S rRNA genes (V6 region) to innate immune responsiveness measured around the time of stool sample collection. This further allowed us to test the hypothesis that regionally distinct gut microbiomes are associated with differential development of systemic immunity. To do so, we employed a sparse partial least squares (sPLS) integrative approach (12) to extract correlations between the microbiome and immune phenotype. We also assessed causality, whether a specific microbiome can drive the development of a particular immune phenotype, via human fecal transplantation into germfree mice. Taken together, this study provided evidence that differences in systemic innate immunity across biogeographically diverse populations correlate with differences in the gut microbiome.

## RESULTS

### Cohort characteristics.

Study participants were recruited between May 2011 and January 2012. Cohort characteristics for the children included in the immune analysis were previously described (6). Stool samples for microbiome analysis were collected from a subset of these children, including 17 Belgian, 32 Canadian, 42 Ecuadorean, and 8 South African children. Blood samples were collected within 10 days of stool samples (see Fig. S1 in the supplemental material). Baseline characteristics differed between the two sites (Table 1). Notably, among the Belgians, only one of 17 subjects was female, while sex was more balanced in the other regional cohorts. Belgians were almost exclusively, and South Africans were exclusively, vaginally delivered, whereas Caesarean delivery was more common in the Canadian and Ecuadorean cohorts. Other differences included anthropometric measurements and the younger average age of recruited Canadians (mean, 1.65 years). Ethnic heterogeneity also varied (Table 2). All children were born and raised at the sites of sample collection, except for a subset of the Belgian children (7%), who were born in African countries or, in one case, Germany. Immune data for these children born outside Belgium were not available.

**TABLE 1:** Cohort characteristics of children included in immune or microbiome analysis

Characteristic^*a*^	Value or information				*P* value^*b*^
	Belgium	Canada	Ecuador	South Africa	
Child characteristics					
*n*	21	33	43	21	
Sex (*n* [%])					
Female	1 (4.8)	16 (48.5)	28 (65.1)	11 (52.4)	<0.001
Male	20 (95.2)	17 (51.5)	15 (34.9)	10 (47.6)	
Missing (no.)	0	0	0	0	
Delivery mode (*n* [%])					
Caesarean	2 (9.5)	16 (48.5)	9 (20.9)	0 (0.0)	<0.001
Vaginal	19 (90.5)	17 (51.5)	34 (79.1)	21 (100.0)	
Missing	0	0	0	0	
Age (mo) (median [IQR])	25.00 (21.00, 27.00)	18.00 (18.00, 20.00)	26.00 (25.00, 26.00)	24.00 (23.00, 24.00)	<0.001
Missing (no.)	1	0	0	0	
Gestational age (wks) (median [IQR])	39 (38, 40)	39 (38, 40)	39 (38, 40)	38 (37, 40)	0.389
Missing (no.)	0	0	9	0	
Birthweight (g) (median [IQR])	3,095 (2,935, 3,480)	3,280 (3,062, 3,610)	3,250 (2,991, 3,647)	3,030 (2,740, 3,300)	0.092
Missing (no.)	3	0	17	0	
WAZ (median [IQR])	0.22 (−0.10, 1.63)	0.32 (−0.30, 1.04)	−0.37 (−0.98, 0.04)	−0.46 (−1.16, 0.07)	<0.001
Missing (no.)	1	0	0	1	
WLZ (median [IQR])	−0.04 (−1.07, 0.81)	0.33 (−0.33, 0.80)	0.22 (−0.41, 0.65)	0.06 (−0.56, 0.32)	0.448
Missing (no.)	1	0	0	1	
HAZ (median [IQR])	1.36 (0.59, 1.85)	0.27 (−0.50, 1.07)	−1.01 (−1.50, −0.30)	−1.27 (−2.07, −0.03)	<0.001
Missing (no.)	1	0	1	0	
Maternal age (yrs) (median [IQR])	33 (28, 36)	35 (33, 38)	26 (21, 30)	25 (22, 28)	<0.001
Missing (no.)	0	0	0	6	
Breastfeeding					
Ever breast fed					
Data collected	No	Yes	Yes	Yes	
Yes (*n* [%])	NA^*c*^	33 (100.0)	42 (100.0)	20 (100.0)	
Missing	NA	0	1	1	
Time since weaning (months)					
Data collected	No	Yes	Yes	Yes	
Yes (median [IQR])	NA	5 (0, 12)	13 (10, 16)		0.001
Missing	NA	0	1	21	
Breastfeeding duration					
Data collected	No	Yes	Yes	No	
Mo (median [IQR])	NA	14 (6, 18)	13 (10, 16)	NA	0.872
Currently breastfeeding					
Data collected	No	Yes	No	No	
No (*n* [%])	NA	24 (72.7)	NA	NA	
Yes (*n* [%])	NA	9 (27.3)	NA	NA	

a WLZ, weight-for-length Z-score; WAZ, weight-for-age Z-score; HAZ, height-for-age Z-score.

b Chi-square test for categorical variables, Kruskal-Wallis test for continuous variables with more than two classes, Wilcoxon rank-sum test for continuous variables with two classes.

c NA, not applicable.

**TABLE 2:** Ethnic background of study participants included in either microbiome or immune analysis

Category	Belgium		Canada		Ecuador		South Africa	
	Description	No. (%)	Description	No. (%)	Description	No. (%)	Description	No. (%)
Ethnic background	African	6 (28.6)	Chinese	3 (9.1)	Latin American	43 (100)	African	4 (19.0)
	African/Arab	2 (9.5)	Filipino	1 (3.0)			Mixed race	16 (76.2)
	Arab	7 (33.3)	Latin American	3 (9.1)			White-Caucasian	1 (4.8)
	Metis/White-Caucasian/Arab	1 (4.8)	White-Caucasian	21 (63.6)				
	South Asian	1 (4.8)	White-Caucasian/Chinese	3 (9.1)				
	White-Caucasian	1 (4.8)	White-Caucasian/Filipino	1 (3.0)				
	White-Caucasian/Arab	1 (4.8)	White-Caucasian/South Asian	1 (3.0)				
	Unknown	2 (9.5)						
Country of birth	Belgium	13 (61.9)	Canada	33 (100)	Ecuador	43 (100)	South Africa	21 (100)
	Congo	2 (9.5)						
	Germany	1 (4.8)						
	Mauritania	1 (4.8)						
	Morocco	3 (14.3)						
	Uganda	1 (4.8)						

10.1128/mBio.03079-20.2 FIG S1 Time between blood draw and stool sample collection. Days between blood and stool sample collection for Belgian, Canadian, Ecuadorean, and South African children for whom both immune and microbiome data were available. Negative values denote stool sample collected prior to blood sample. Missing data: Belgium, *n* = 6; no missing data points for Canadian, Ecuadorean, or South African cohorts. Download FIG S1, TIF file, 0.4 MB. Copyright © 2021 Amenyogbe et al. 2021 Amenyogbe et al. This content is distributed under the terms of the Creative Commons Attribution 4.0 International license.

### Child stool microbiome was strongly impacted by country of birth.

The 16S amplicon libraries yielded a total of 4,030 OTUs after quality filtering and binning at a 97% similarity threshold. Sequencing depth did not differ significantly among cohorts (data not shown).

### (i) Alpha diversity.

**FIG 1:**
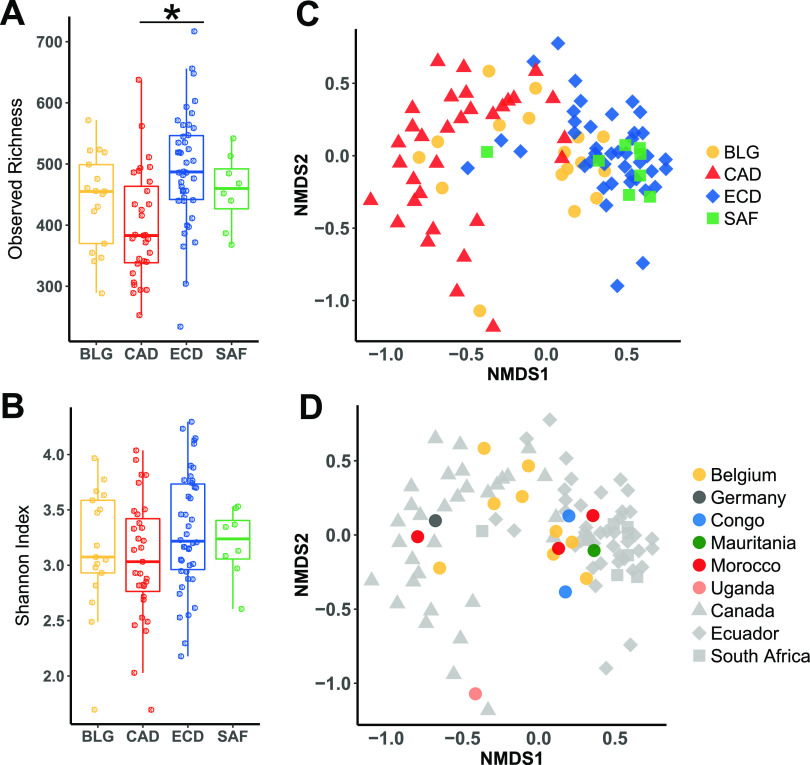
Alpha and beta diversity of child fecal microbiomes. Alpha diversity in cohorts from each country determined using observed richness (A) or Shannon Diversity (B). Statistics: Kruskal-Wallis test with Dunn’s posttest, *P* values adjusted with the Benjamini-Hochberg method. *, adjusted *P* < 0.05. (C) Beta diversity based on nonmetric multidimensional scaling (NMDS) coded according to country of residence. (D) Beta diversity based on NMDS with Belgian children coded according to country of birth.

We first sought to understand whether there was a difference in alpha diversity among the four cohorts by calculating the observed richness or the Shannon index to estimate diversity. The Canadian children harbored significantly fewer species than Ecuadorean children (Fig. 1A). Shannon diversity of fecal microbiota did not differ among cohorts (Fig. 1B). Based on linear regression, host demographic factors did not correlate with Shannon diversity across the four cohorts, but there were some correlations within individual cohorts. Delivery mode correlated with diversity in the Canadian cohort (see Fig. S2A). Maternal age correlated with diversity in Ecuadorean and Canadian cohorts (Fig. S2B). Because Canadian mothers who delivered by Caesarean section (C-section) were older than those that delivered vaginally, we used multiple linear regression, which showed that maternal age remained significantly associated with diversity, while the delivery mode did not. Interestingly, maternal age was positively correlated with diversity in Ecuadoreans but negatively correlated in Canadians. When data from both cohorts were combined, the younger maternal age range of Ecuadoreans and older range of the Canadians revealed a significant quadratic relationship (method; *P* < 0.05, *R*^2^ = 0.09), with both the youngest and oldest mothers having children with lower diversity (Fig. S2C).

10.1128/mBio.03079-20.3 FIG S2 Interactions between host factors and fecal microbiome alpha diversity. (A) Relationship of diversity and delivery mode in Canadian and Ecuadorian cohorts. (B) Correlation of diversity and maternal age in Canadian and Ecuadorian cohorts. (C) Quadratic relationship between diversity and maternal age in combined Canadian and Ecuadorian cohorts. Download FIG S2, TIF file, 0.3 MB. Copyright © 2021 Amenyogbe et al. 2021 Amenyogbe et al. This content is distributed under the terms of the Creative Commons Attribution 4.0 International license.

### (ii) Beta diversity.

We found that the microbiomes differed substantially for Canadian versus Ecuadorian and South African populations (Fig. 1C). Of note, Belgian microbiomes were distributed across both groups, with the Belgian African-born subgroup (comprised of subjects born in different African countries) more often clustering with South Africans and Ecuadorians (Fig. 1D).

Forward selection-based analysis to determine the contribution of demographic variables (sex, delivery mode, gestational age, maternal age, and anthropometric measurements) to the explanation of community composition resulted in cohort (country of origin) as the sole variable of importance (adjusted *R*^2^ = 0.11, *P* = 0.002), with no other demographic variables contributing significantly to community composition. Because the effect of cohort could potentially mask effects of demographic variables within each cohort, we performed distance-based redundancy analysis (dbRDA) and ordiR2step for these variables in each cohort separately. We found that weight-for-length Z-score at the time of sampling was significant in explaining community composition in Canadian (adjusted *R*^2^ = 0.031, *P* = 0.031) and South African (adjusted *R*^2^ = 0.17, *P* = 0.020) children. No other host factors were significant for any other cohort. Thus, the demographic variables measured did not have major associations with microbiome composition.

### (iii) Microbiome taxonomic composition.

**FIG 2:**
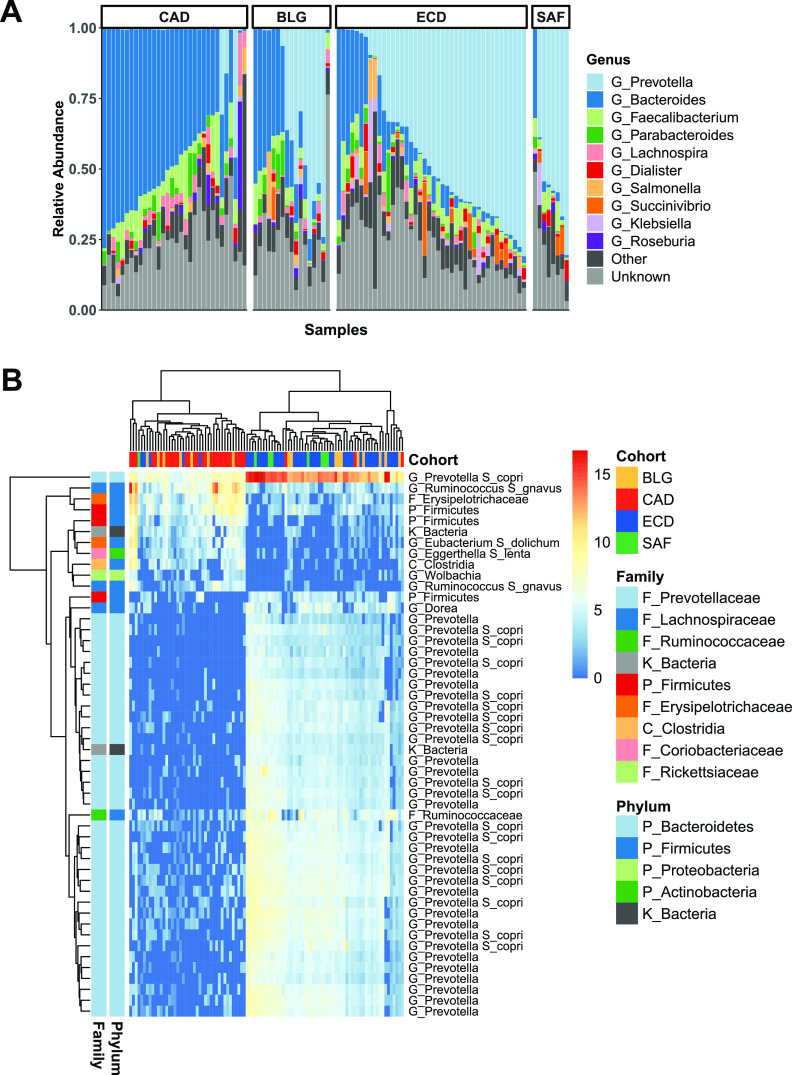
Taxonomic compositions of child fecal microbiomes. (A) Top 10 most abundant genera in members of cohorts from each country ordered by relative abundance of *Bacteroides*. (B) Heat map showing normalized abundance of the top 50 taxa differentially abundant across all cohorts determined via DESeq2 analysis (adjusted *P* < 0.01) and further selected by multivariate PLS-DA. Hierarchal clustering of subjects according to abundance profiles of taxa (top) and of taxa according to their abundance profiles across subjects (left).

Taxonomic compositions of microbiomes reflected commonly identified human taxa, with *Prevotella*, *Bacteroides*, *Faecalibacterium*, *Lachnospira*, and *Dialister* being the top 5 most abundant genera (Fig. 2A). Most individuals were dominated by either *Prevotellaceae* or *Bacteroidaceae*.

We tested for associations between abundances of individual OTUs and cohort membership with the DESeq2 likelihood ratio test and found 442 OTUs differentially abundant among the cohorts. These OTUs were ranked for their capacity to discriminate cohorts using partial least squares discriminant analysis (PLS-DA). The top 50 OTUs selected by PLS-DA discriminated Canadians and Belgian-born Belgians versus Ecuadoreans, South Africans, and African-born Belgians (Fig. 2B). However, these 50 OTUs did not discriminate South Africans from Ecuadoreans or discriminate Belgians from any other cohort. Selected OTUs enriched in the Ecuadorean and South African clusters were almost exclusively members of *Prevotella*. A smaller subset of OTUs enriched in the Canadian cluster included a diverse range of genera, mostly belonging to the *Firmicutes* (including *Ruminococcus*, *Clostridia*, and unclassified *Firmicutes*). *Prevotella* OTUs were very rare in Canadians, present at high abundances in only 3 of 32 children.

### PRR ligand-specific responses associated with each cohort.

**FIG 3:**
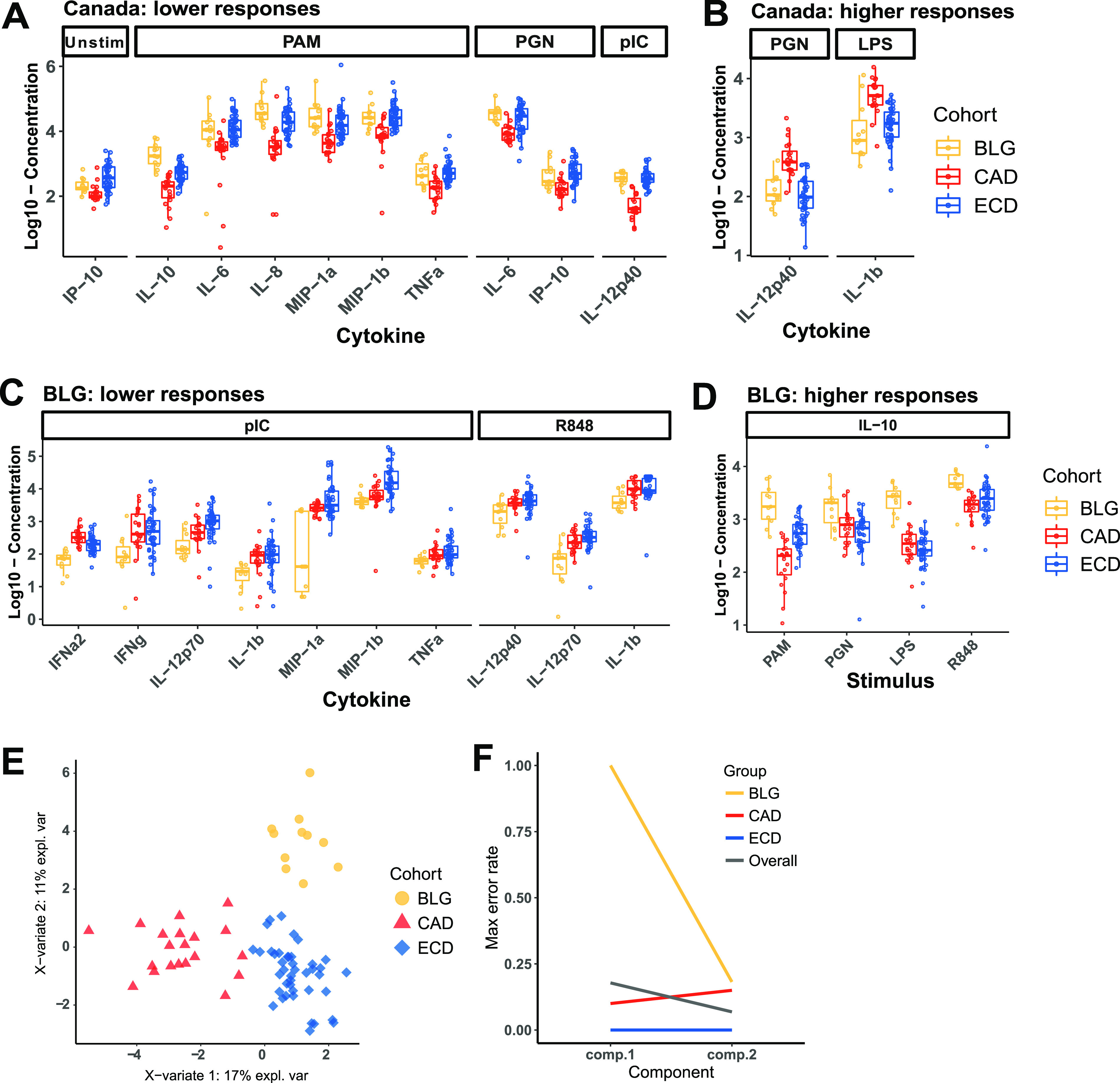
Discriminatory cytokines for Canadian and Belgian children selected by sPLS-DA. (A and B) Discriminatory features in component 1 with lower or higher responses in Canadians. (C and D) Discriminatory features in component 2 with lower or higher responses in Belgians. (E) sPLS-DA ordination of children based on cohort-defining cytokines. (F) Maximum error rates overall and per class show that Belgian (BLG), Canadian (CAD), and Ecuadorian (ECD) data are classified with error rates of 18%, 10%, and 0%, respectively, with a minimum overall error rate of 6.8%.

Cytokine responses to PRR stimulation were published previously (6), most notably showing that children from South Africa were distinct in profoundly underresponding to every PRR agonist except for peptidoglycan (PGN; stimulates TLR2 and nucleotide-binding oligomerization domain-containing protein 1/2 [NOD1/2]), based on univariate tests and principal-component analysis (PCA). However, additional albeit more subtle differences existed among the other cohorts. sPLS-DA identified discriminatory cytokine responses among Canadian, Belgian, and Ecuadorean children. Canadians were classified largely by lower responses to PAM3CYSK4 (PAM; TLR2 agonist) stimulation than Ecuadoreans and Belgians (Fig. 3A and B), while Belgians were classified largely by lower responses to endosomal PRR agonists, poly(I·C) (stimulates TLR3) and R848 (stimulates TLR7/8) (Fig. 3C and D). Ecuadoreans did not have lower or higher responses to any PRR stimulation and were thus classified by their exclusion from the other two cohorts. The cytokine response signatures allowed us to classify each cohort with an error rate of 25% or less with only two sPLS-DA components (Fig. 3E and F).

### TLR responsiveness correlated with distinct microbiome features.

**(i) Findings across cohorts.** Integration analysis was performed using study participants with both microbiome and complete immune data available: 8 Belgian, 19 Canadian, 41 Ecuadorean, and 8 South African children. Initial integration of OTU and cytokine data using all available subjects yielded a very poor correlation structure between the two data sets. Because South African immune profiles were highly distinct from those of all other cohorts, we hypothesized that the extreme phenotype of this cohort correlated poorly with those of the other cohorts. To this end, we performed sPLS integration using data from Belgian, Canadian, and Ecuadorean children only. The features that were selected whether South African children were included or not overlapped substantially. However, the covariance of selected features was weaker when South African children were included (see Fig. S4).

10.1128/mBio.03079-20.4 FIG S3 sPLS integration performed with all children or only Belgian, Canadian, and Ecuadorean children. Percentages of significantly covarying cytokines to OTUs selected (A) or OTUs significantly covarying with selected cytokines (B) by both models. Lines denote 30% cutoff for selected feature retention among all children (blue) and BLG, CAD, and ECD children (red). (C) Heat map showing correlations between selected OTUs and cytokines that correlate with at least 30% of features of the complementary data type. (D) Network of cytokine-OTU correlations between selected cytokines and OTUs. Edge colors denote Pearson correlation strengths. Correlations between *Bacteroides* OTUs and cytokine responses to PAM among all children (E) and BCG, CAD, ECD children only (F). *r* statistics and *P* values computed via Pearson correlation. Download FIG S3, TIF file, 0.9 MB. Copyright © 2021 Amenyogbe et al. 2021 Amenyogbe et al. This content is distributed under the terms of the Creative Commons Attribution 4.0 International license.

10.1128/mBio.03079-20.5 FIG S4 Belgian cohort-specific integrations. Correlation circle plot (A) and network showing correlation structure between selected OTUs and cytokines (B). Network edge colors represent Pearson correlation strength. (C) Examples of sPLS-selected OTUs correlating to cytokines in response to PAM (TLR2/6 stimulation). Statistics: Pearson correlation strength and significance. Canadian cohort-specific integrations. Correlation circle plot (A) and network showing correlation structure between selected OTUs and cytokines (B). Network edge colors represent Pearson correlation strength. (C) Examples of sPLS-selected OTUs correlating to cytokines in response to PAM (TLR2/6 stimulation). Statistics: Pearson correlation strength and significance. Ecuadorean cohort-specific integrations. Correlation circle plot (A) and network showing correlation structure between selected OTUs and cytokines (B). Network edge colors represent Pearson correlation strength. (C) Examples of sPLS-selected OTUs correlating to cytokines in response to PGN (TLR2/6 and NOD stimulation). Statistics: Pearson correlation strength and significance. South African cohort-specific integrations. Correlation circle plots (A and B) and network showing correlation structure between selected OTUs and cytokines (C). Network edge colors represent Pearson correlation strength. (D) Examples of sPLS-selected OTUs correlating to cytokines in response to PGN (TLR2/6 and NOD stimulation). Statistics: Pearson correlation strength and significance. Download FIG S4, PDF file, 0.7 MB. Copyright © 2021 Amenyogbe et al. 2021 Amenyogbe et al. This content is distributed under the terms of the Creative Commons Attribution 4.0 International license.

**FIG 4:**
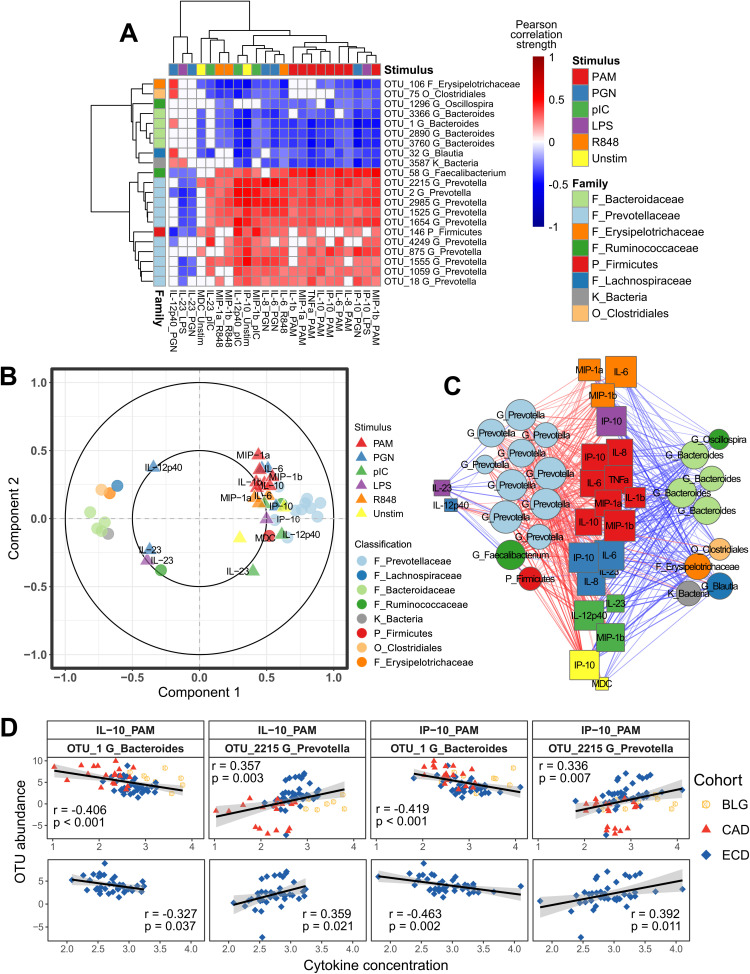
Cytokine-microbe correlations across multiple cohorts. (A) Heat map showing correlations between selected OTUs and cytokines, with hierarchical clustering of OTUs based on cytokine correlation profiles (left) and hierarchical clustering of cytokines based on OTU correlation profiles (top). Correlation circle plot (B) and network showing correlation structure among selected OTUs and cytokines (C). In the circle plot, circles indicate OTUs and triangles indicate cytokines. Network edge colors denote Pearson correlation strength, and nodes are sized according to number of connections. (D) Selected correlations between *Bacteroides* OTU_1 and cytokine responses to PAM3CYSK4 stimulation (IL-10 and IP-10) and positive correlations between *Prevotella* OTU_2215 and the same responses, as observed across Belgian, Canadian, and Ecuadorian cohorts (top) and in only the Ecuadorian cohort (bottom).

The sPLS model including Belgian, Canadian, and Ecuadorean children was thus utilized for further analyses. Covaried OTUs and cytokines were selected along the first sPLS component (Fig. 4A). The selected features from both data sets were dominated by negative correlations between *Bacteroides* OTUs and cytokine responses to PAM (TLR2) stimulation and positive correlations between *Prevotella* OTUs and the same responses (Fig. 4A to C). These associations were also significant in the Ecuadorean cohort alone (Fig. 4D). Interleukin 6 (IL-6), IL-8, and interferon gamma-induced protein 10 (IP-10) responses to PGN (TLR2 and NOD1/2) and macrophage inflammatory protein 1 alpha (MIP-1α) and MIP-1β responses to endosomal TLR stimulation followed the same pattern. IL-23 responses to both PGN and lipopolysaccharide (LPS) were selected for their distinct relationships to the selected OTUs, correlating negatively with *Prevotella* but not with *Bacteroides*.

**FIG 5:**
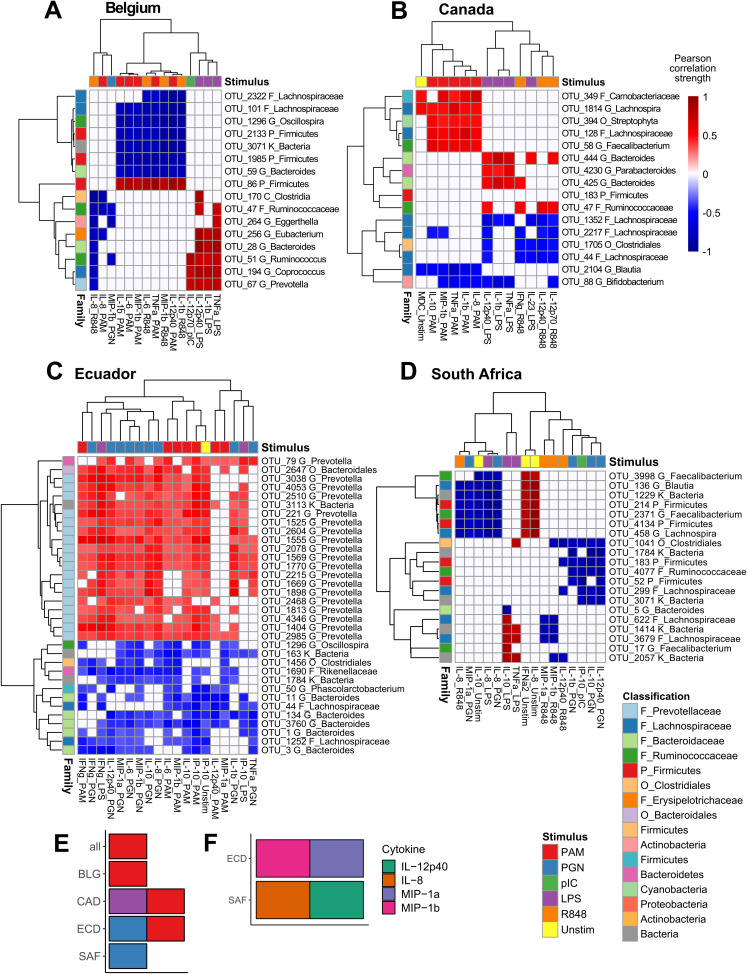
Cohort-specific integrations selected distinct cytokine-OTU relationships. Heat maps show Pearson correlations between sPLS-selected OTUs and cytokines in cohorts from Belgium (A), Canada (B), Ecuador (C), and South Africa (D). Hierarchical clustering as for Fig. 4. OTU legend colors are assigned at the family level for the top 8 represented families (or highest level of classification) in all analyses combined. The remaining OTUs are colored according to phylum. (E) Overrepresented TLR ligand responses among all samples and within each cohort separately. (F) Overrepresented cytokine responses within cohorts from Ecuador and South Africa.

**(ii) Findings within cohorts.** Unique OTU-cytokine correlations were additionally identified within individual cohorts (Fig. 5; Fig. S4). Cytokine responses to PAM were overrepresented features in sPLS models for Belgium, Canada, and Ecuador separately and combined (Fig. 5E). Cytokine overrepresentation in models were rare and only included MIP-1α and MIP-1β among Ecuadoreans, and IL-8 and IL-12p40 among South Africans (Fig. 5F). The only bacterial family overrepresented in any cohort model was the *Prevotellaceae* for both the three combined cohorts and Ecuador separately.

Among the Belgians, cytokine responses to PAM stimulation and to R848 (TLR7/8) stimulation all negatively correlated with *Firmicutes*, including *Lachnospiraceae* and *Oscillospira*, and cytokine responses to LPS positively correlated with *Firmicutes*, including *Clostridia* and *Ruminococcus* (Fig. 5A and S4A).

The Canadian cohort was dominated by proinflammatory and Th17-supporting cytokine responses to LPS (including IL-1β, tumor necrosis factor alpha [TNF-α], IL-23, and IL-12p40), and Th1 responses to R848 (including gamma interferon [IFN-γ], IL-12p40, and IL-12p70) correlated with several *Lachnospiraceae* and *Bacteroides* OTUs (Fig. 5B and S4B). Additionally, cytokine responses to PAM positively correlated with a diverse subset of *Firmicutes*.

Among Ecuadoreans, cytokine responses to both PAM and PGN stimulation were overrepresented and correlated with multiple *Prevotella* and *Bacteroides* OTUs (Fig. 5C and S4C). Also, production of MIP-1α and MIP-1β correlated with diverse bacterial taxa.

South African children were the only ones for which responses to PAM were not overrepresented. However, responses to PGN were overrepresented, as were cytokines IL-12p40 and IL-8, in response to multiple ligands. These correlated almost exclusively to *Firmicutes* (Fig. 5D and S4D).

**(iii) Host factors did not associate with host microbiome-immune correlations.** At the time of enrollment, seven Canadian children and one Ecuadorean child included in microbiome-immune integration were still being breastfed (see Fig. S5A). We identified a negative relationship between duration since weaning and OTUs belonging to the *Lachnospiraceae* family and the *Roseburia* genus (Fig. S5B). These associations were not present among Ecuadorian children (Fig. S5C and D). No correlations were identified between any breastfeeding factors and immune responses either in block sPLS or in univariate assessment of each stimulus-cytokine pair individually. The remainder of host factors demonstrated sparse relationships to either OTUs or cytokines unique to either Canadian or Ecuadorean children, including associations between delivery mode, sex, weight-for-length Z-scores (WLZ), and weight-for-age Z-scores (WAZ), among Canadian children (see Fig. S7A and B, examples in E to G) and between delivery mode and maternal age for Ecuadorean children (Fig. S7C and D, examples in H to I).

10.1128/mBio.03079-20.6 FIG S5 Associations between breastfeeding and the microbiome. (A) Duration of breastfeeding for Canadian and Ecuadorean children. (B) Heat map indicating significant indicating significant correlations between time since breastfeeding, breastfeeding duration, and microbes along the second model component. Correlations between time since breastfeeding and *Roseburia* (C) and *Lachnospiraceae* (D) among Canadian and Ecuadorean children. Download FIG S5, TIF file, 0.4 MB. Copyright © 2021 Amenyogbe et al. 2021 Amenyogbe et al. This content is distributed under the terms of the Creative Commons Attribution 4.0 International license.

Among Canadian and Ecuadorean children together, maternal age, height-for-age Z-scores (HAZ), WAZ, and time since breastfeeding were found to covary with *Bacteroides*, *Prevotella*, and responses to TLR2 stimulation (see Fig. S6A). Given that the host factors showing strongest associations also differed between these cohorts, we determined the correlation strength of these relationships among Canadian and Ecuadorean children separately, finding that globally, these associations were no longer significant (Fig. S6B and C, with specific examples in D and E). Thus, demographic factors, stool microbiome composition, and innate immunity did not correlate across multiple cohorts.

10.1128/mBio.03079-20.7 FIG S6 Block-sPLS integration of microbiome, immune, and microbiome data among Canadian and Ecuadorean children. Heat maps depicting correlation strengths between demographic factors and host features selected by sPLS among both cohorts (A), Canadian children only (B), and Ecuadorean children only (C). Plots depicting significant correlations between maternal age, HAZ, *Bacteroides*, and *Prevotella* OTUs among all children (D), but not significant among Canadian or Ecuadorean children separately (D and E). Download FIG S6, TIF file, 1.1 MB. Copyright © 2021 Amenyogbe et al. 2021 Amenyogbe et al. This content is distributed under the terms of the Creative Commons Attribution 4.0 International license.

10.1128/mBio.03079-20.8 FIG S7 Block-sPLS integration of microbiome, immune, and microbiome data among Canadian and Ecuadorean children separately. Heat maps indicating significant correlations between host factors, OTUs, and cytokines for the first and third model components for Canadian children (A and B) and between delivery mode along the first and maternal age along the second model components for Ecuadorean children (C and D). Boxplots indicating significant correlations between delivery mode (E), child sex (F), and WAZ and WLZ scores (G) and sPLS-selected OTUs and cytokines among Canadian children. Boxplots indicating significant correlations between delivery mode, OTUs, and responses to R848 stimulation (H) and plots indicating negative correlations between maternal age and *Prevotella* OTUs (I) among Ecuadorean children. Download FIG S7, TIF file, 1.1 MB. Copyright © 2021 Amenyogbe et al. 2021 Amenyogbe et al. This content is distributed under the terms of the Creative Commons Attribution 4.0 International license.

### Fecal transplant dictates immune phenotype of germfree mice.

**FIG 6:**
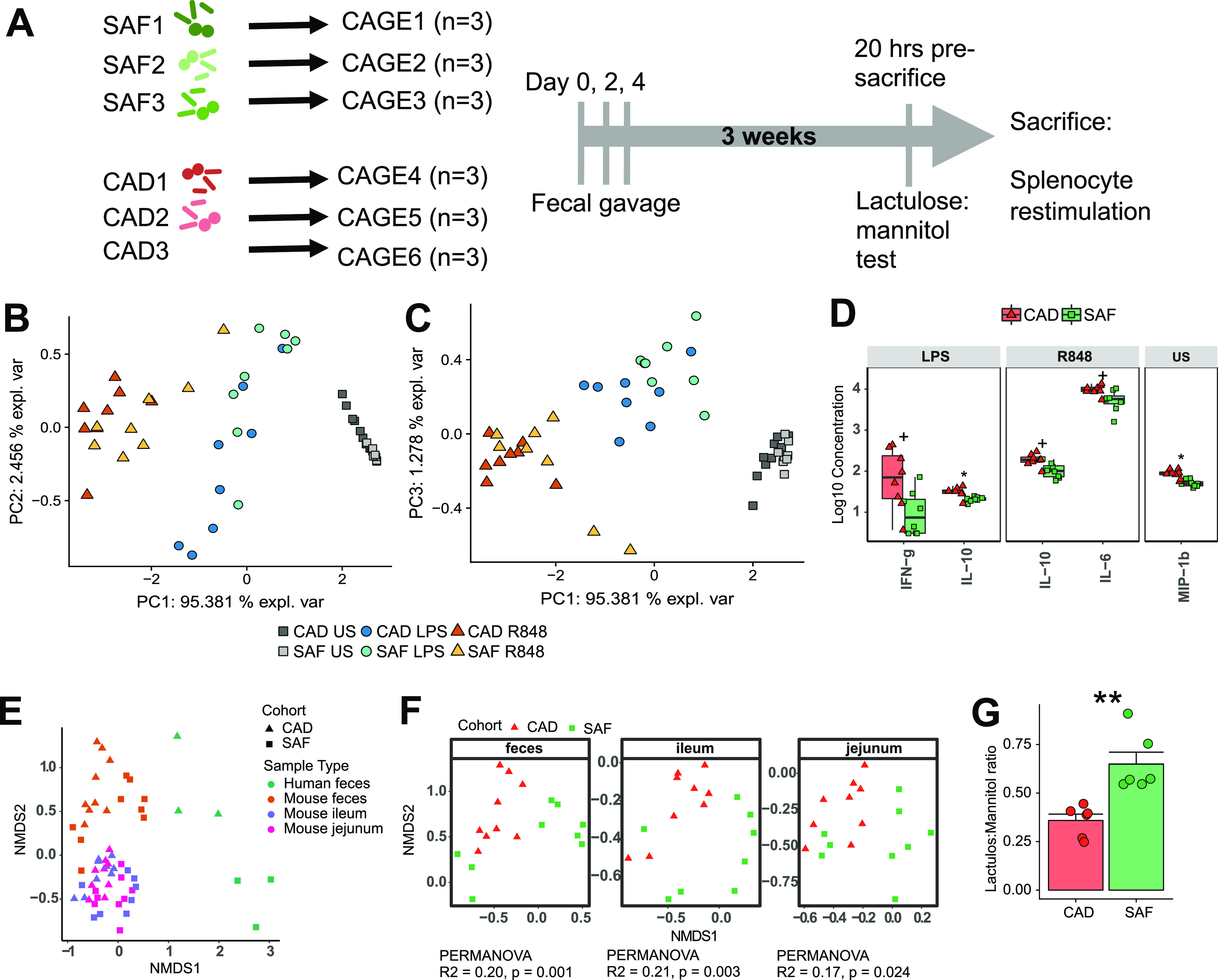
Human fecal transplantation into germfree mice recapitulated immune signatures in human donors. (A) Schematic of experimental design. Principal-component analyses of cytokine responses by murine splenocytes showing principal component 1 versus 2 (B) and 1 versus 3 (C), demonstrating clustering by stimulus and by transplant donor cohort. (D) Cytokine responses to TLR stimulation that significantly differed between mice gavaged with Canadian versus South African child stools. Statistical analyses: Wilcoxon rank-sum test, *P* values adjusted via Benjamini-Hochberg method with *q* < 0.1 considered significant; *, *q* < 0.05; +, q < 0.1 (all nominally significant). Boxplots indicate medians with first and third quartiles (25% to 75%); whiskers extend no further than 1.5 × interquartile range (IQR) from the hinge. (E) NMDS of Bray-Curtis distance showing similarity of microbiomes in mouse feces, ileum, and jejunum and human feces. (F) NMDS of Bray-Curtis distance showing community composition of microbiomes in mouse feces, Ileum, and jejunum separately, with statistics indicating variance contributed by stool donor cohort. (G) Lactulose/mannitol ratios of mice given South African (SAF) versus Canadian (CAD) child stools; the groups are significantly different (*P* < 0.01, Student's *t* test).

Mouse models of human fecal transplantation are a potentially useful tool to dissect host-microbiome relationships *in vivo* (13, 14). In a proof-of-principle experiment, we directly tested whether human gut microbiota used to colonize germfree mice could induce differences in systemic immune phenotypes similar to those observed in the human donors. We compared South African versus Canadian microbiomes for their potential effects on splenocyte responses to TLR stimulation in germfree mice (Fig. 6A). Principal-component analysis of recipient mouse cytokine responses to PRR stimulation demonstrated that the type of stimulus primarily determined the response (principal component 1), as was observed within the human data (6). However, within each stimulus, cohort-specific clustering was evident for responses to both R848 (PC1 versus PC2) (Fig. 6B) and LPS (PC1 versus PC3) (Fig. 6C). In mice inoculated with South African feces versus Canadian feces, IFN-γ and IL-10 responses to LPS and IL-10 and IL-6 responses to R848 were significantly suppressed (Fig. 6D). Responses to proinflammatory cytokines TNF-α and MIP-1β were similar between the groups, while IL-23 and IFN-α2 were not produced in this assay (see Fig. S8A). Overall, mice inoculated with South African feces mounted lower cytokine responses than those inoculated with Canadian feces, as was observed in the corresponding donor children.

10.1128/mBio.03079-20.9 FIG S8 (A) Cytokine responses to TLR stimulation in germfree mice inoculated with Canadian or South African feces. Statistical analysis: Wilcoxon rank-sum test, *P* values adjusted using the Benjamini-Hochberg method. *, *q* ≤ 0.05; +, *q* ≤ 0.1; ns, q > 0.1. OTUs differentially abundant between mice inoculated with CAD versus SAF child fecal microbiomes. Differentially abundant OTUs in the jejunum (B), ileum (C), and feces (D). (E) Four OTUs enriched in SAF sample-inoculated mice that were enriched in South African donor stools. (F) Two OTUs enriched in CAD sample-inoculated mice that were enriched in Canadian donor stools. All OTUs presented in panels D and E were significant after correction using the DESeq2 test. Boxplots indicate medians with first and third quartiles (25% to 75%). Whiskers extend no further than 1.5 × IQR from the hinge. Download FIG S8, TIF file, 0.9 MB. Copyright © 2021 Amenyogbe et al. 2021 Amenyogbe et al. This content is distributed under the terms of the Creative Commons Attribution 4.0 International license.

We assessed the intestinal and fecal microbiomes of recipient mice at the end of the experiment alongside re-extracted DNA from human donor stools (Fig. 6E). The microbiomes of the mice were distinct from those of their respective source feces. However, within each tissue and the feces, the microbiomes were distinct between mice with the different fecal sources (Fig. 6F). Furthermore, the small intestinal barrier integrity was significantly lower between mice inoculated with South African feces than those inoculated with Canadian feces (Fig. 6G). A subset of the differentially abundant OTUs in the original human samples were also found in the mice (Fig. S8B to D). Of the OTUs differentially abundant in both the human donors and the mice, six consistently distinguished the treatment groups in the ileum, jejunum, or feces. Among these, four OTUs, belonging to the genera, *Alistipes*, *Odoribacter*, and *Prevotella*, and to the family *Rikenellaceae*, were enriched in the South African human donor stools and in the mice inoculated with those stools (Fig. S8E). The remaining two OTUs, belonging to the genus *Clostridium*, were enriched in the Canadian human donor stools and in the mice inoculated with those stools (Fig. S8F).

## DISCUSSION

Given the ability of the host microbiome to modulate innate immunity as well as the known geographical variability of both gut microbiomes and innate immune phenotypes, it is surprising that a correlation between the two was not previously assessed. Also missing is a mechanistic understanding of how the geographically distinct microbiomes contribute to host immune differences. Robust correlations from human studies are therefore needed to inform mechanistic work using animal models. Here, we provide evidence from humans on the biogeography of the relationship between host microbiome and systemic immunity and a proof of concept supporting the existence of a causal relationship between them.

While this study did not incorporate data pertaining to diet and lifestyle, the observed differences in stool microbiome compositions are consistent with surveys of the gut microbiome in similar environments in terms of resource availability and diet. For example, children living in a rural environment in Burkina Faso, where diets are rich in complex carbohydrates and soluble fiber but low in animal fats and proteins, were colonized by *Prevotella*, while children living in westernized environments such as Italy and the United States, where animal products are a major part of the diet, were dominated by *Bacteroides* (2, 3). Some of these differences may be driven by lifestyles associated with urbanization. Urban dwellers in both Nigeria and Burkina Faso have microbiomes more closely resembling those of residents of industrialized urban centers than those of rural dwellers from the same countries (15, 16), again with greater relative abundance of *Bacteroides* than *Prevotella* in the urban dwellers. However, the Belgian children included in this study all lived in an urban environment, yet stool microbiomes of some Belgians with African heritage were dominated by *Prevotella* and clustered with Ecuadorean and South African children. Relative abundance of *Bacteroides* among Ecuadoreans was variable and did not correlate with breastfeeding. Hence, diet alone is unlikely to explain all global patterns of stool microbiome composition we observed. Notably, *Bacteroides* were well represented within the Ecuadorean children, even in individuals where *Prevotella* abundance was high. However, *Prevotella* were not detected in Canadian children dominated by *Bacteroides*, suggesting that those environments may have been unfavorable for *Prevotella* colonization.

We found that the demographic factors we measured did not have strong associations with the compositions of the children’s microbiomes. In the Canadian and Ecuadorean cohorts, we did find a quadratic relationship between maternal age and microbial diversity, with lower diversity associated with both younger and older mothers. The lack of this association within the Belgian and South African cohorts may reflect the lower sample sizes in these cohorts, although it is possible that it reflects a region-specific effect of maternal age on child microbiomes. Both extremes of maternal age have been associated with increased risk for adverse birth outcomes (17), stunting at 2 years of age, and altered glucose metabolism in adulthood (18). The possibility that the microbiome is involved in such outcomes associated with maternal age warrants further investigation.

Using multi-omic integration, we identified correlations between the host microbiome and systemic immune responses, both within and across cohorts. Most notably, we found that higher cytokine responses to TLR2 were associated with a greater relative abundance of *Prevotella* and a lower relative abundance of *Bacteroides* in the Belgian, Canadian, and Ecuadorean cohorts. We did not provide direct evidence that *Prevotella* modulates TLR2 responsiveness or identify any correlations with health outcomes. *Bacteroides* and *Prevotella* have been shown to modulate mucosal immune responses through TLR2. Prevotella copri induces a more robust proinflammatory cytokine response from human dendritic cells in a TLR2-dependent manner (19, 20). These effects on dendritic cells link *Prevotella*-rich gut dysbioses in humans to rheumatoid arthritis (21, 22) and periodontal disease (23). Conversely, Bacteroides fragilis sphingolipid polysaccharide A (PSA) also signals through TLR2, stimulating dendritic cells to produce IL-10, contributing to an anti-inflammatory environment systemically (24) and in the mucosa (25, 26). *Prevotella* species have also been associated with positive health outcomes. Research has revealed substantial diversity among the *Prevotella* genus (27), and many of the OTUs identified as *Prevotella* in our data were not classified at the species level. Gut microbiomes dominated by *Prevotella* have been associated with increased levels of short-chain fatty acids (SCFAs) in a rural African setting (2) and with high-fiber diets among adults living in westernized nations (28, 29).

Our study revealed closer associations of the TLR2 response pathway with the host microbiome than other PRRs. The selection of microbial taxa other than *Bacteroides* and *Prevotella* in cohort-specific networks suggests other immunomodulatory relationships that have not yet been experimentally evaluated. Finally, not all cohort-specific immune responses correlated with fecal microbiomes. For example, we did not find any relationships between increased IL-10 responsiveness among the Belgian children and their gut microbiomes. Thus, there are likely additional environmental or genetic determinants of systemic innate immune phenotypes that we did not capture.

Breastmilk modulates the gut microbiome through several mechanisms, including variations in human milk oligosaccharide (HMO) composition (30). HMO compositions vary by geographical region and lifestyle factors (31, 32). Among microbial taxa selected by microbiome-immune integration, *Bifidobacteria*, *Prevotella*, and *Bacteroides* are able to use HMOs for growth (33, 34). However, we were not able to identify effects of time since breastfeeding on immune responsiveness in either Canadian or Ecuadorean children, and among Canadian children, time since breastfeeding was only associated with levels of *Lachnospira* and *Rothia* but not with cytokine responses, even though seven of 19 children were still breastfeeding. With this, breastfeeding was not a likely contributor to the host immune-microbiome correlations we observed. However, given the small sample size applied to this analysis and that these data were only available for Canadian and Ecuadorean children, identifying more subtle relationships between breastfeeding and immune responsiveness or gut microbiota composition was not possible, and these findings do not preclude the existence of such relationships.

Innate immune phenotypes of South African children were previously described by us to be highly distinct from those of the other cohorts (6), while our present study found their gut microbiomes to be indistinguishable from those of Ecuadorean children. Notably, fecal gavage of germfree mice resulted in mouse immune phenotypes consistent with those of the respective donor children. South African donors induced strikingly lower cytokine responses favoring Th1 and Th17 development. However, proinflammatory cytokine responses were unaffected. Thus, the microbiome-induced mouse phenotypes were partly, but not completely, in agreement with those previously reported for the children (6). Importantly, we did not identify a causal mechanism or a specific component of the microbiome responsible for the observed effects on immune phenotype in germfree mouse recipients. Hence, these results provide biological plausibility that must be further explored using animal models and validated in human cohorts. While this extreme-phenotype approach likely did not capture all relevant interactions, these *in vivo* data support the existence of a causal relationship between human gut microbiomes and systemic immune function.

There are limitations to this study worth noting. The major limitations of this study were the sample size and the statistical power to detect associations between host microbiome and immune phenotypes. Similar studies that identified robust associations between gut microbiome, immune phenotype, and host demographic factors among adults included >500 adults (8). This limitation does not negate the associations that were identified but may have caused us to overlook weaker associations. This study also suffered from unequal sample numbers among the four cohorts. With this, the absence of significant findings in some instances, especially the lack of associations with host factors, may have been due to the lack of statistical power to detect subtle relationships. Belgian children, uniquely, were almost all male, while child sex was more balanced for the other three cohorts. However, given that child sex was not a significant contributor to microbiome community composition at the other three sites and child sex was not found to contribute to cohort-specific cytokine responses in our previous findings (6), it is unlikely that the sex bias of the Belgian cohort influenced the integration results. We limited the number of OTUs retained for integrated analysis to roughly 5% of those identified among the four cohorts. Also, especially for Ecuadorean children, differences in time between blood sample and stool sample collection may have influenced the results. We also did not analyze the fecal metagenome, which has been shown to associate with systemic immunity in previous work (8). While the cohorts are referred to by their countries of recruitment, the enrolled subjects are not always representative of the overall populations or resource availability in those countries. The germfree mouse experiments were not conducted in a germfree facility, which may have contributed to the divergence of engrafted mouse microbiomes from the original inocula.

### Conclusion.

This study provided supporting evidence to link geographically distinct immune phenotypes to gut microbiomes and identified a predominant association between systemic cytokine responses to TLR2 stimulation and stool microbiome composition. We also provide supporting evidence via human fecal transplantation in germfree mice showing that the human host microbiome can induce changes to systemic immunity. Monitoring the gut microbiome and immune system ontogeny along with well-defined clinical outcomes (e.g., infections or vaccine responses) in larger cohorts will further the understanding of geographic differences in those clinical outcomes.

## MATERIALS AND METHODS

### Ethics statement.

All research involving humans was conducted according to principles in the Declaration of Helsinki and approved by the University of British Columbia ethics board under protocol number H11-01423. Each study site obtained ethical approval separately from their research institutions. Informed consent was obtained from primary guardians for children involved in this study. Research involving animals was conducted under ethical approval from the University of British Columbia animal care committee under protocol number A13-0265.

### Recruitment of study participants.

The recruitment of the four cohorts of children of approximately 2 years of age was previously described (6). Study participants were recruited from ongoing collaborative studies or healthy child cohorts at each of the four sites. Canadian children were recruited at the BC Children’s Hospital in Vancouver (35). Belgian children were enrolled in a birth cohort enrolling St Pierre Hospital in Brussels and included mostly healthy male children presenting for a routine circumcision. Ecuadorean children were enrolled in the ECUAVIDA birth cohort in Quininde (36), and South African children were enrolled in a prospective birth cohort at the Tygerberg Academic Hospital in Cape Town (37, 38). Participants were only included in the study if the child was considered healthy based on medical history, and they were excluded if they met one or more of the following criteria: significant chronic medical condition, immune deficiency, immunosuppression by disease or medication, cancer, bone marrow or organ transplantation, receipt of blood products within 3 months, bleeding disorder, major congenital malformation, genetic disorder, or born to HIV-positive mothers.

### Innate immune phenotyping.

Innate immune phenotyping for these cohorts was previously described and published (6). Briefly, 3 to 5 ml of peripheral blood was drawn per participant. Whole blood was then stimulated with the following PRR agonists: PAM3CYSK4 (PAM, stimulates TLR2), poly(I·C) (stimulates TLR3), lipopolysaccharide (LPS; stimulates TLR4), resiquimod (R848; stimulates TLR7/8), peptidoglycan (PGN; stimulates both TLR2 and nucleotide-binding oligomerization domain-containing protein 1/2 [NOD1/2]), and medium alone. Whole blood was stimulated for 24 h, and supernatants were analyzed for the following cytokines measured using the Luminex multiplex assay (Luminex, Upstate/Millipore Flex kit system): IFN-α2, IFN-γ, CXCL10, IL-12p70, IL-12p40, IL-6, TNF-α, IL-1β, CXCL8, CCL3, CCL4, and IL-10.

### Child fecal microbiome analysis.

Human stool microbiome composition was determined using amplicon sequencing targeting the V6 region of the 16S rRNA gene. Stool samples were collected within the same month as blood samples for each child, stored at −80°C, and transported to the Vancouver laboratory on dry ice. Total DNA was extracted from all samples within 1 month of arrival to the laboratory using the Qiagen QIAamp DNA stool minikit (Qiagen catalog number 51504). PCR and DNA sequencing were according to previously described protocols and rationale for amplicon sequencing targeting the V6 region of the 16S rRNA gene (39). Further details on microbiome analysis can be found in Text S1 in the supplemental material.

10.1128/mBio.03079-20.1 TEXT S1 The supplement includes detailed information for human stool sample collection, DNA extraction, and V6-16S amplicon sequencing (library preparation and sequencing). For germfree mouse experiments, supplementary methods include further details describing mice used for the experiment, human fecal transplantation, and measuring the lactulose to mannitol ratio in mouse urine samples. For the immunological experiments performed for germfree mice, further details of splenocyte stimulation with TLR agonists and measurements of supernatant cytokines are supplied. Further details are also given for V4-16S amplicon sequencing of germfree mouse intestinal and fecal samples. For statistical analysis, steps taken to prepare amplicon sequencing and immune data for analysis and detail for all analyses performed throughout the manuscript are supplied. Download Text S1, DOCX file, 0.04 MB. Copyright © 2021 Amenyogbe et al. 2021 Amenyogbe et al. This content is distributed under the terms of the Creative Commons Attribution 4.0 International license.

### Germfree mouse model of human fecal transplantation.

To test whether divergent immune phenotypes of South African children could be causally linked to their gut microbiomes, we performed a proof-of-principle experiment whereby male germfree Swiss-Webster mice were gavaged with stools from either Canadian or South African male children. Stool samples from only male children were selected to match the sex of experimental animals, of which only males were available. Three weeks after gavage, we compared the splenocyte cytokine responses to TLR stimulation and assessed their gut barrier integrity by using the lactulose-mannitol test. Mouse fecal and intestinal microbiomes at the end of the experiment were measured via 16S amplicon sequencing. DNA from the donor human stools was re-extracted, PCR amplified, and sequenced on the same sequencing run as the mouse samples. The experimental design is shown in Fig. 6A, and further experimental details can be found in Text S1.

### Statistical analyses.

**(i) Microbiome analysis.** Briefly, we assessed differences in gut microbiomes of children across study sites using measures of alpha diversity (observed richness and Shannon index) and beta diversity. Differences in microbiome community composition were further explored by identifying discriminatory OTUs among cohorts using univariate (DESeq2 [40]) and multivariate (sparse partial least squares discriminant analysis [sPLS-DA] [41]) approaches. We also conducted exploratory analyses to determine whether host factors captured in our study (sex, delivery mode, anthropometric measurements, gestational age, birthweight, and maternal age) were associated with differences in either alpha or beta diversity of the gut microbiome. Further details can be found in Text S1.

**(ii) Cytokine response signatures among cohorts.** We used sPLS-DA to identify cytokine signatures that distinguish Belgian, Canadian, and Ecuadorean children in a multivariate space and calculated the classification accuracy for each site.

**(iii) sPLS integration of cytokine, microbiome, and demographic data.** To uncover potential gut microbiome-host immune interactions, we examined the joint multivariate structure of gut microbiota compositions and host innate immune responses via sPLS analysis, a method that incorporates variable selection, making it particularly suitable for high-dimensional data sets (41). This analysis was performed to identify both interactions that were robust among all children and interactions specific to individual cohorts. To this end, separate analyses were performed for all cohorts combined and for each cohort separately. Further details can be found in Text S1.

### Data availability.

All sequencing data presented in the manuscript has been deposited at the National Center for Biotechnology Information Sequence Read Archive (NCBI SRA). Sequencing data from stool samples are available under BioProject accessions PRJNA660015 for human stool microbiome and PRJNA662365 for germfree mouse microbiome data sets.

Data presented in the manuscript and accompanying scripts are publicly accessible at https://github.com/nelly-amenyogbe/Global_cohort_microbiome_immun.
