# Supplementary figures and images for: Biogeography of the Relationship between the Child Gut Microbiome and Innate Immune System
Source: mBio. 2021 Jan 12;12(1):e03079-20. doi: 10.1128/mBio.03079-20 (PMC7845628; doi:10.1128/mBio.03079-20)

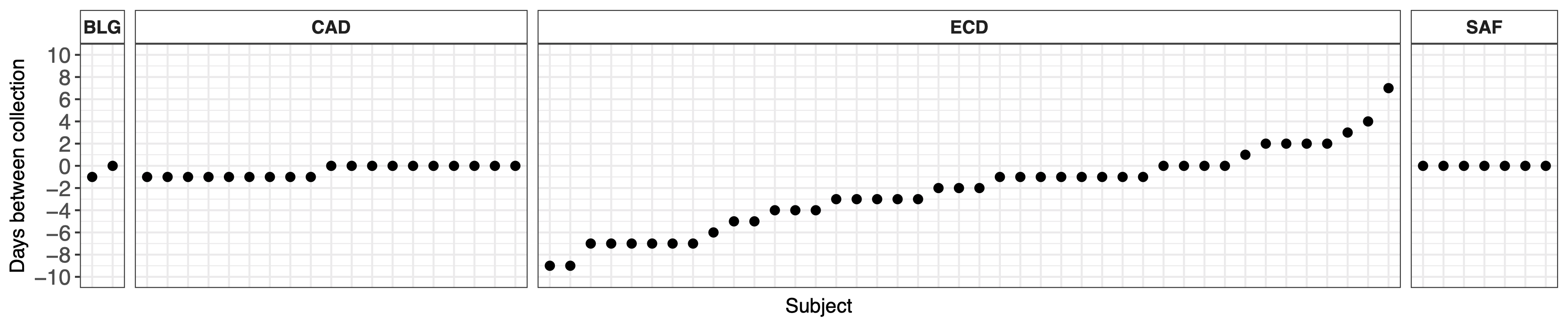

Supplement: FIG S1 [file mBio.03079-20-sf001.tif]

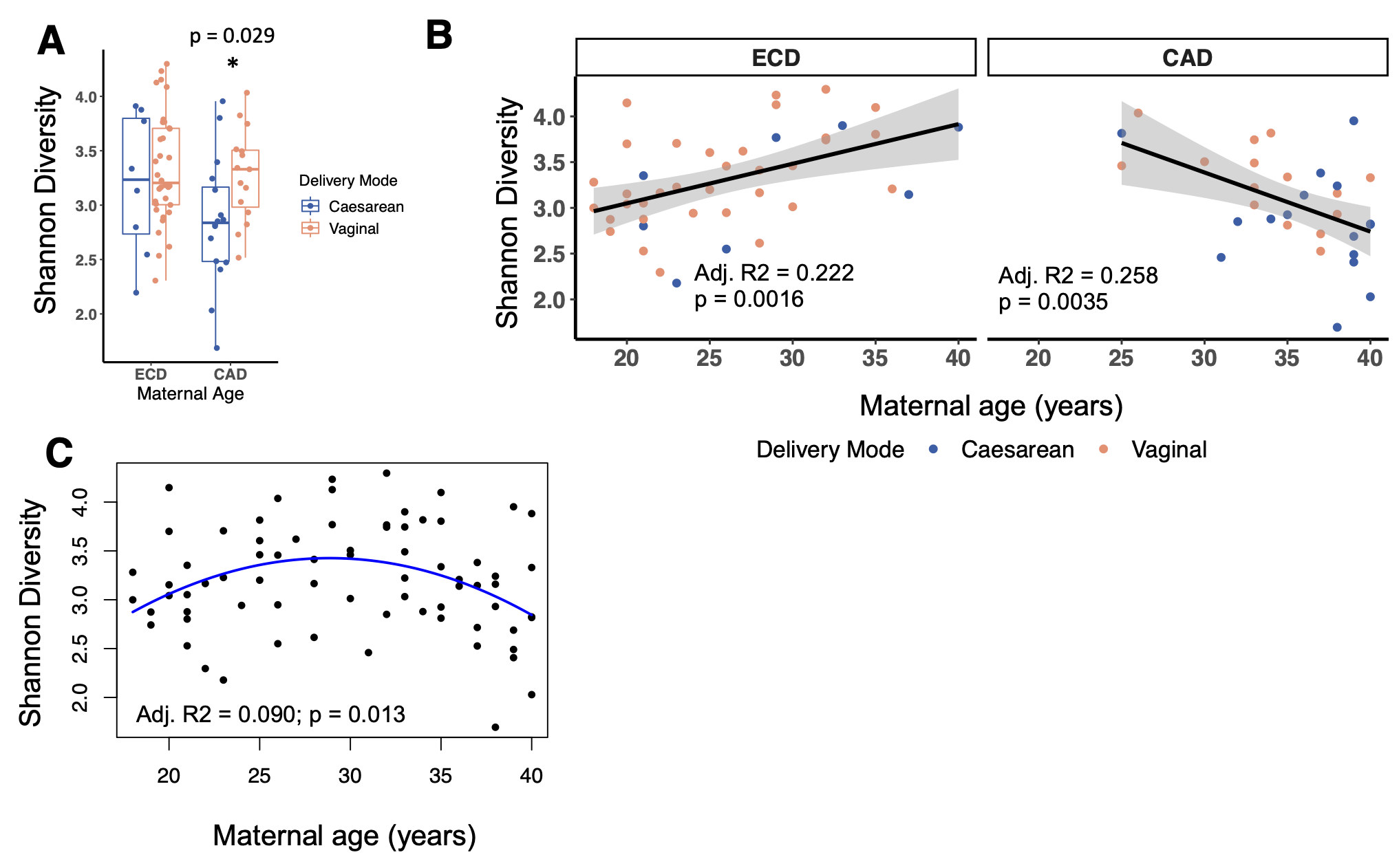

Supplement: FIG S2 [file mBio.03079-20-sf002.tif]

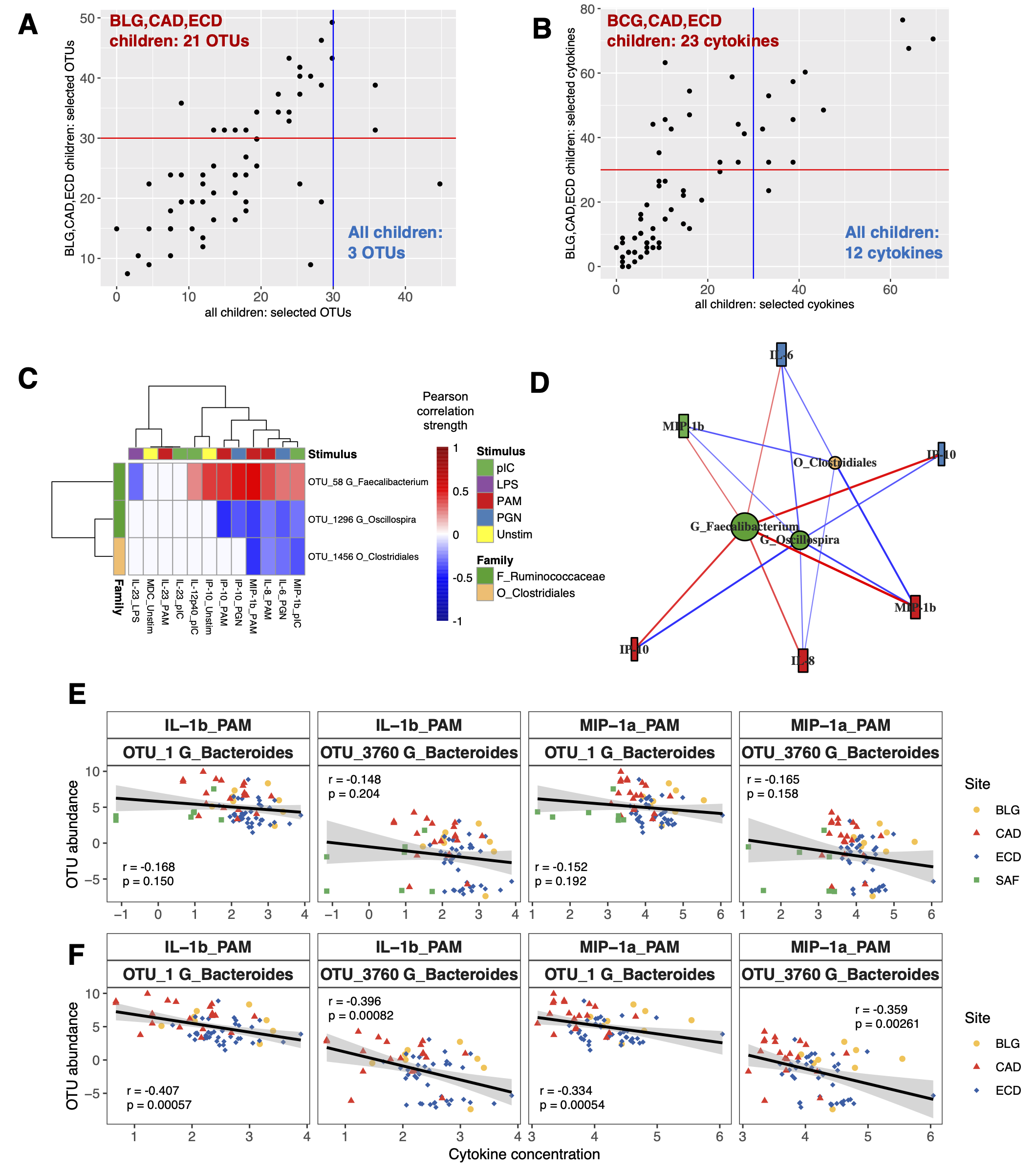

Supplement: FIG S3 [file mBio.03079-20-sf003.tif]

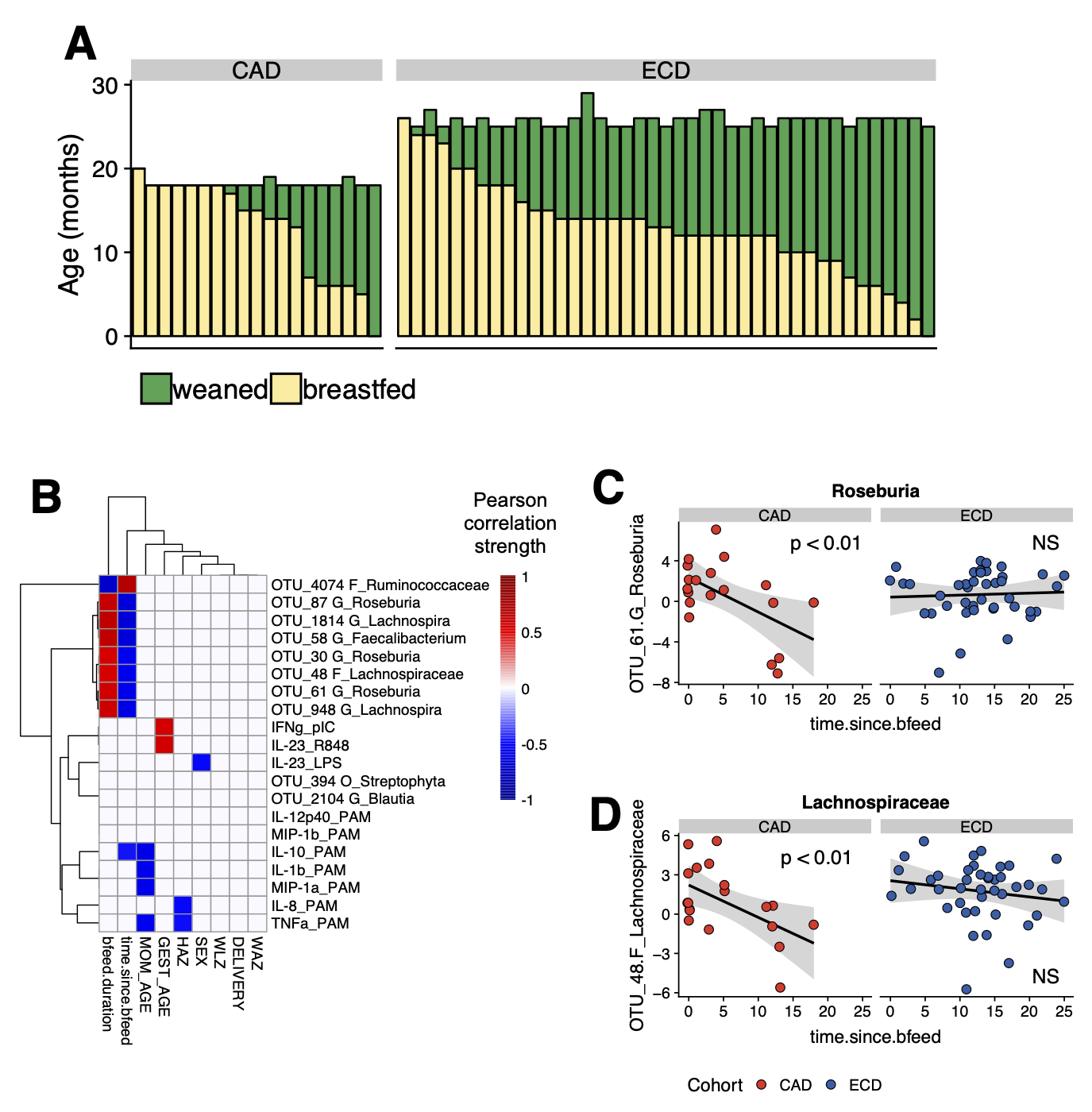

Supplement: FIG S5 [file mBio.03079-20-sf005.tif]

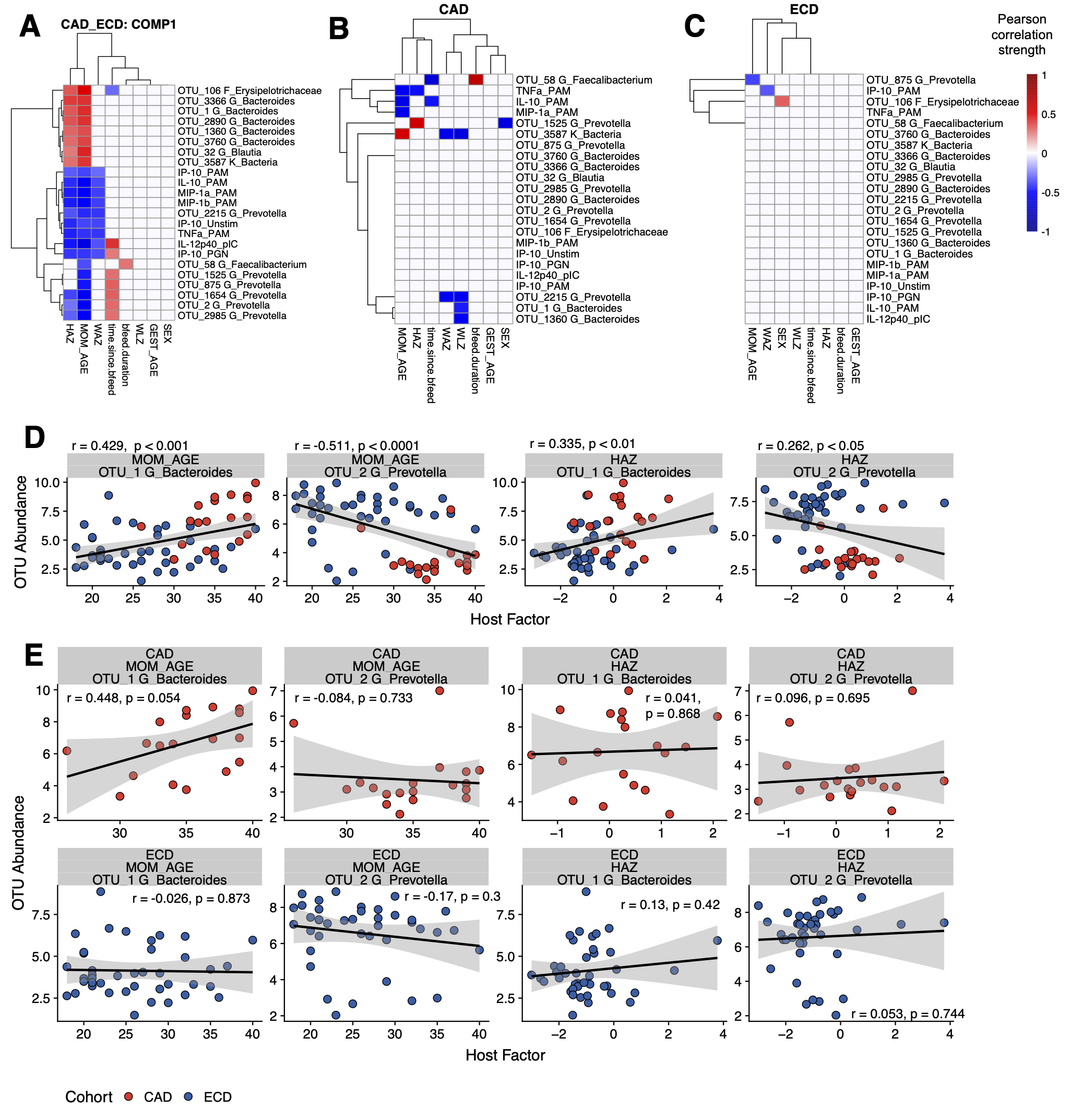

Supplement: FIG S6 [file mBio.03079-20-sf006.tif]

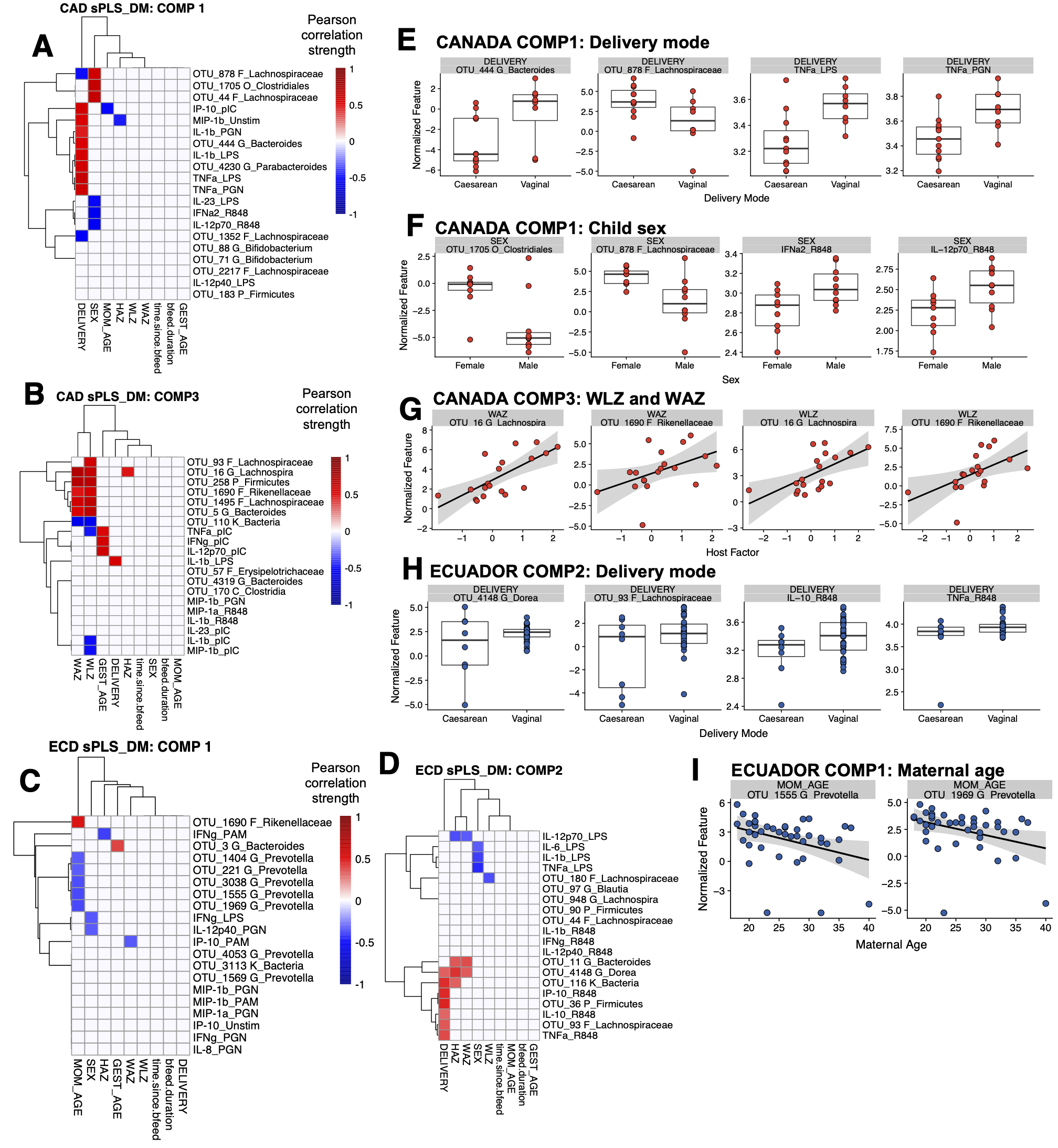

Supplement: FIG S7 [file mBio.03079-20-sf007.tif]

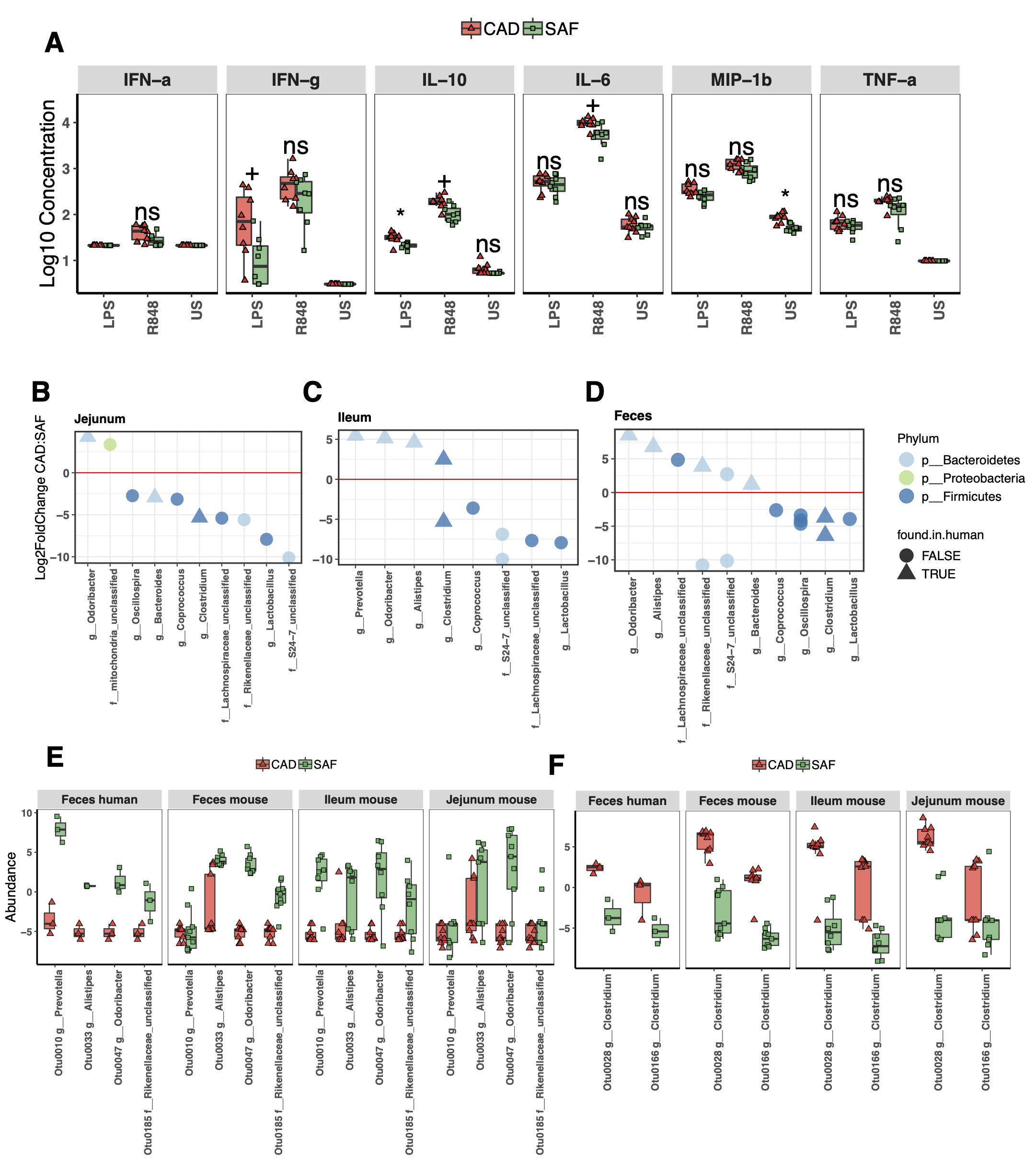

Supplement: FIG S8 [file mBio.03079-20-sf008.tif]
