# Supplementary material for: Biogeography of the Relationship between the Child Gut Microbiome and Innate Immune System
Source: mBio. 2021 Jan 12;12(1):e03079-20. doi: 10.1128/mBio.03079-20 (PMC7845628; doi:10.1128/mBio.03079-20)
Supplement: FIG S4 [file mBio.03079-20-sf004.pdf]

sPLS: Belgium

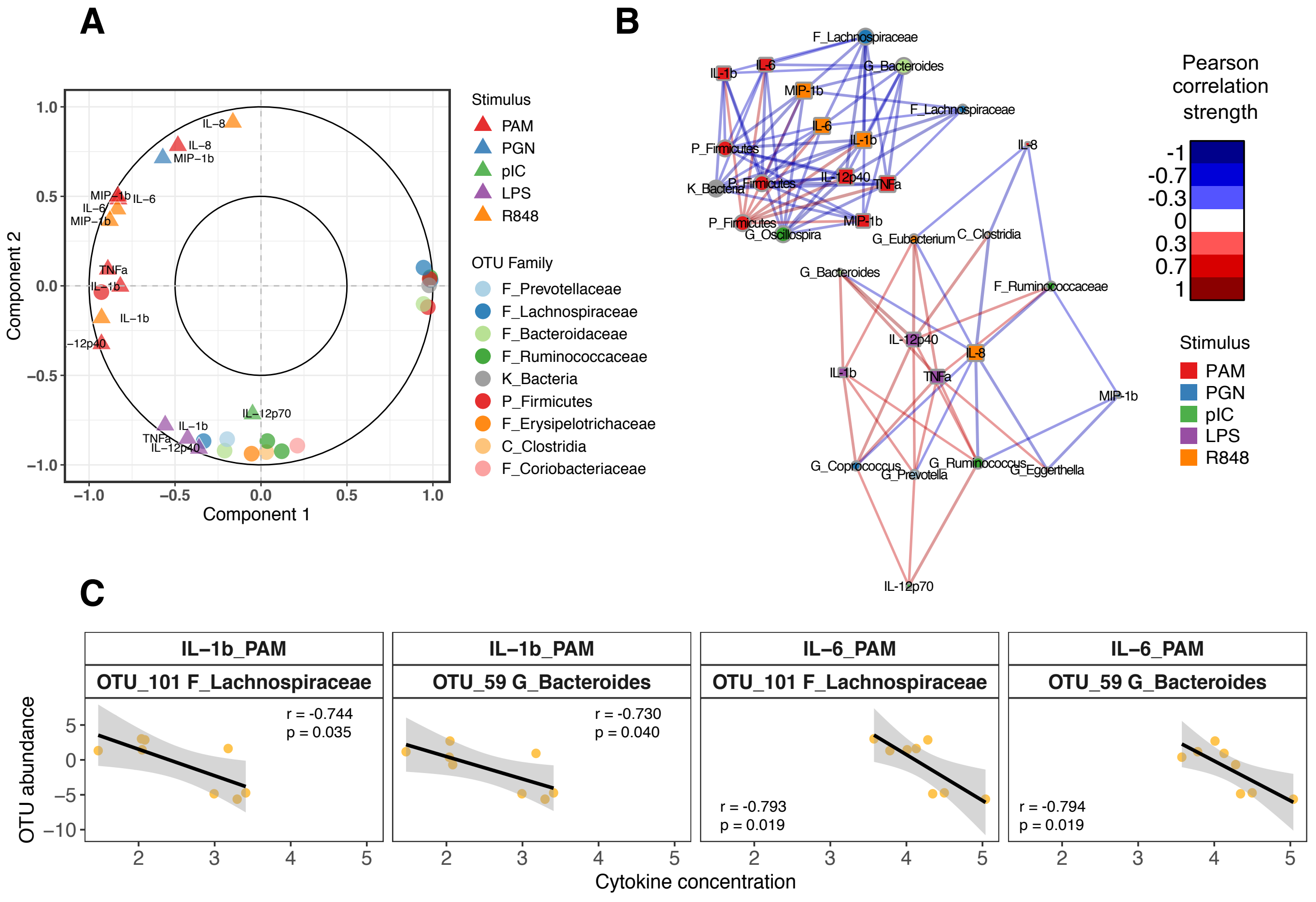

sPLS: Canada

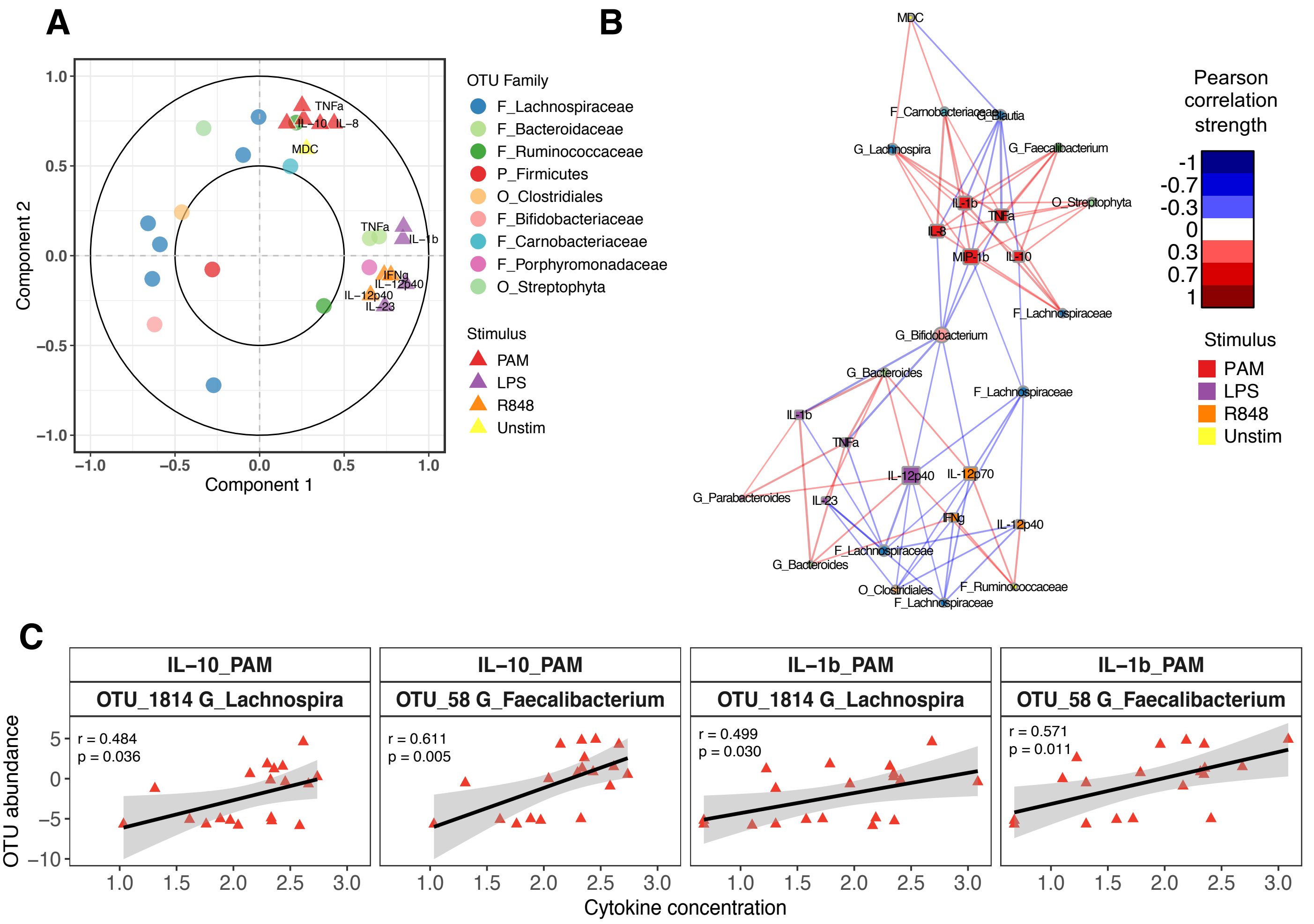
A
B
C
Component 1
Component 2
TNFa
IL−10
IL−8
MDC
TNFa
IL−1b
IFNg
IL−12p40
IL−12p40
IL−23
OTU Family
F_Lachnospiraceae
F_Bacteroidaceae
F_Ruminococcaceae
P_Firmicutes
O_Clostridiales
F_Bifidobacteriaceae
F_Carnobacteriaceae
F_Porphyromonadaceae
O_Streptophyta
Stimulus
PAM
LPS
R848
Unstim
MDC
F_Carnobacteriaceae
G_Blautia
G_Lachnospira
G_Faecalibacterium
IL-1b
O_Streptophyta
TNFa
IL-8
MIP-1b
IL-10
F_Lachnospiraceae
G_Bifidobacterium
G_Bacteroides
F_Lachnospiraceae
IL-1b
TNFa
IL-12p40
IL-12p70
G_Parabacteroides
IL-23
IFNg
IL-12p40
F_Lachnospiraceae
G_Bacteroides
O_Clostridiales
F_Ruminococcaceae
F_Lachnospiraceae
Pearson correlation strength
-1
-0.7
-0.3
0
0.3
0.7
1
Stimulus
PAM
LPS
R848
Unstim
IL−10_PAM
OTU_1814 G_Lachnospira
r = 0.484
p = 0.036
IL−10_PAM
OTU_58 G_Faecalibacterium
r = 0.611
p = 0.005
IL−1b_PAM
OTU_1814 G_Lachnospira
r = 0.499
p = 0.030
IL−1b_PAM
OTU_58 G_Faecalibacterium
r = 0.571
p = 0.011
OTU abundance
Cytokine concentration

sPLS: Ecuador

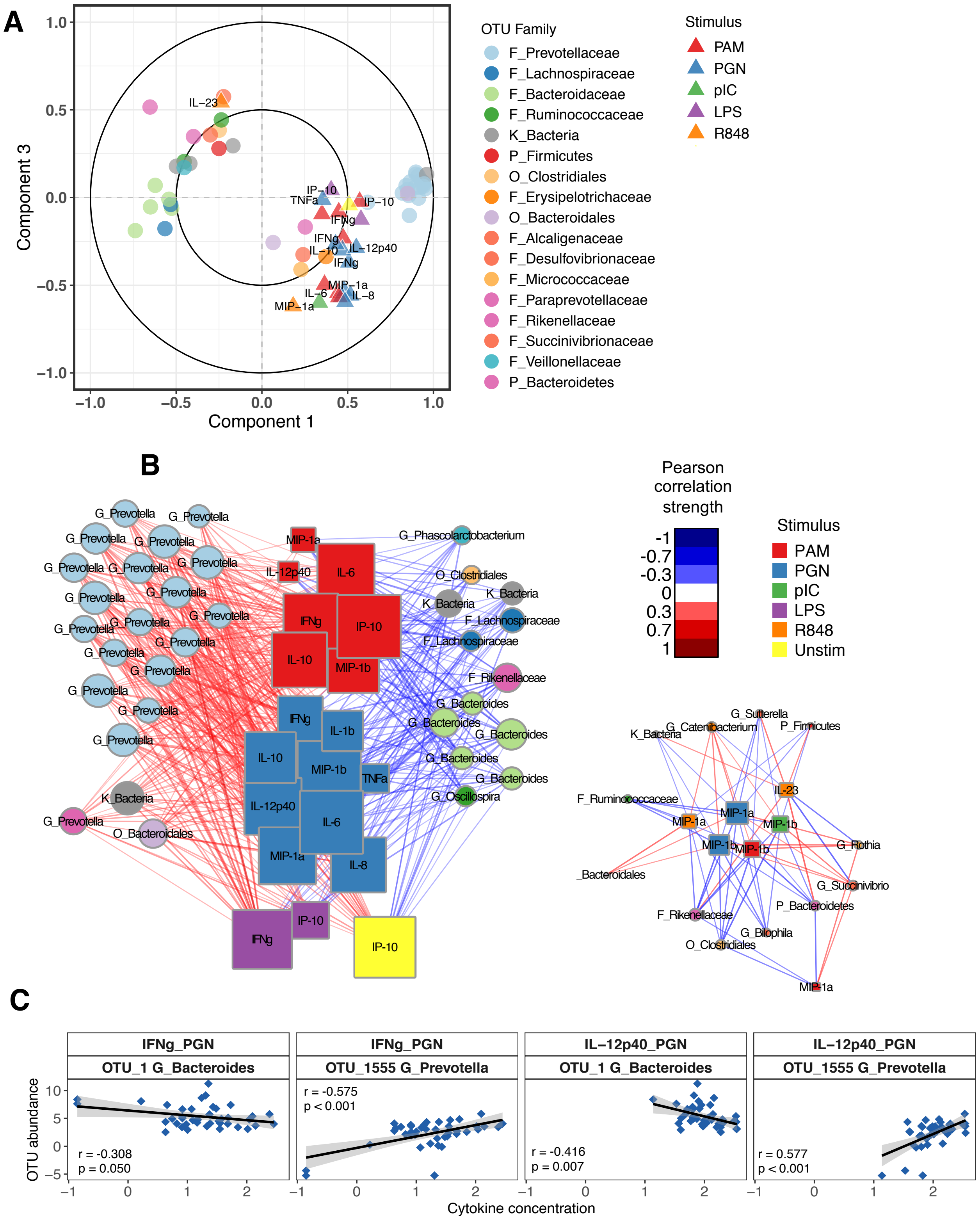

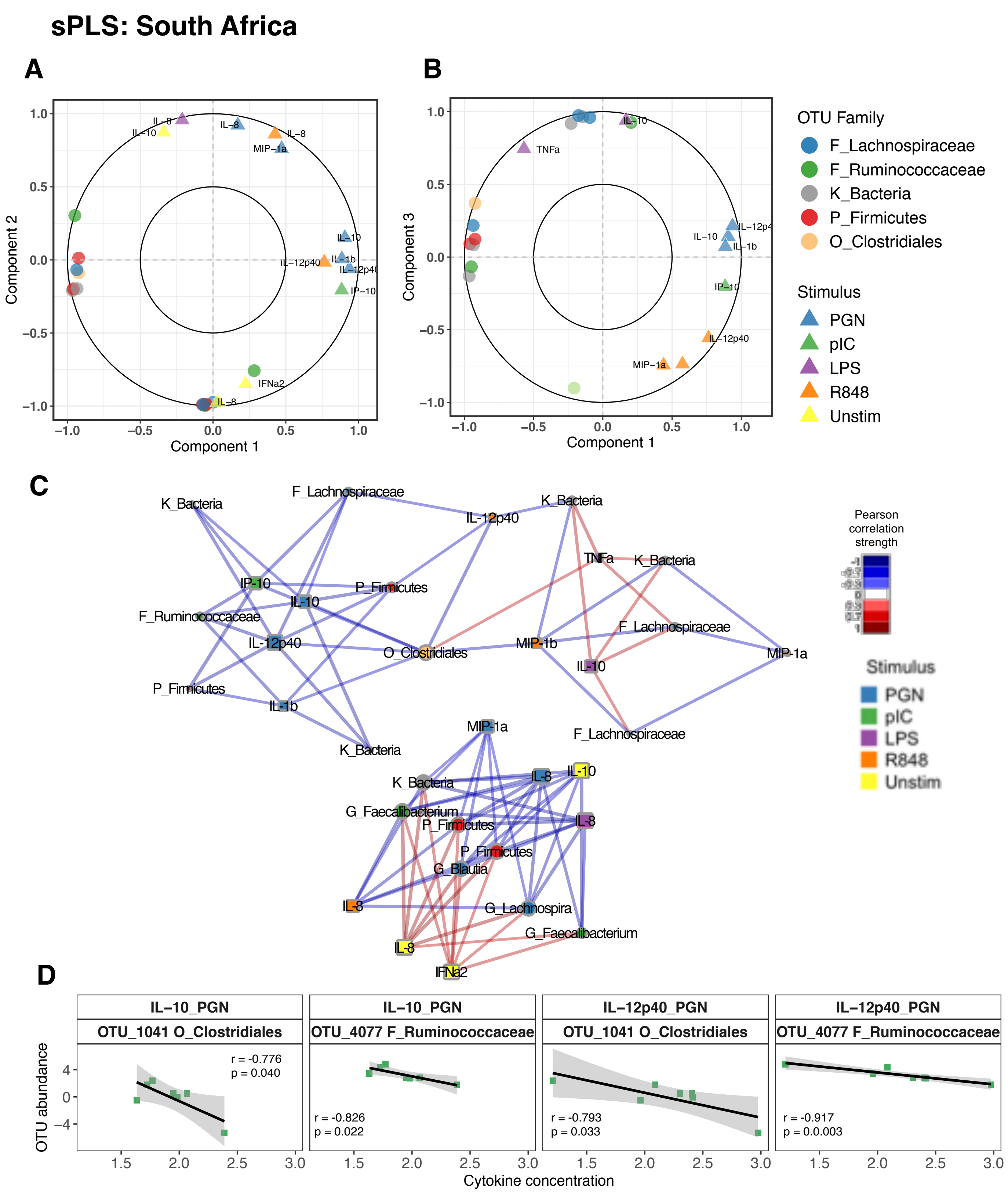

sPLS: South Africa
A
B
C
D
Component 1
Component 2
Component 3
OTU Family
F_Lachnospiraceae
F_Ruminococcaceae
K_Bacteria
P_Firmicutes
O_Clostridiales
Stimulus
PGN
pIC
LPS
R848
Unstim
Pearson correlation strength
IL−10_PGN
OTU_1041 O_Clostridiales
r = -0.776
p = 0.040
IL−10_PGN
OTU_4077 F_Ruminococcaceae
r = -0.826
p = 0.022
IL−12p40_PGN
OTU_1041 O_Clostridiales
r = -0.793
p = 0.033
IL−12p40_PGN
OTU_4077 F_Ruminococcaceae
r = -0.917
p = 0.0.003
OTU abundance
Cytokine concentration
