# Supplementary material for: Biogeography of the Relationship between the Child Gut Microbiome and Innate Immune System
Source: mBio. 2021 Jan 12;12(1):e03079-20. doi: 10.1128/mBio.03079-20 (PMC7845628; doi:10.1128/mBio.03079-20)
Supplement: TEXT S1 [file mBio.03079-20-s0001.docx]

**Biogeography of the relationship between the child gut microbiome and innate immune system**

**Supplementary Methods**

**16S Amplicon Sequencing: Human samples**

**Stool sample collection.** Parents were instructed to collect soiled diapers at home into clean containers and keep at 4^o^C. Samples were delivered to the laboratory within 24 hours of the bowel movement. At the laboratory, samples were aliquoted into 2-ml screw-top tubes except for samples from Vancouver, which were stored in 50-ml conical tubes. All aliquots were stored at -80^o^C within 24 hours of sample receipt. Stool samples from Brussels, Cape Town, and Quininde were shipped to the central study site (Vancouver, Canada) on dry ice via World Courier, Inc. with a temperature monitor to ensure all samples remained below -80^o^C during transport. Samples were stored at -80^o^C in Vancouver for no longer than 24 months prior to DNA extraction.

**Stool DNA extraction.** Total DNA was extracted from 180 – 220 mg stool using the QIAamp DNA stool mini kit (Qiagen Cat. No. 51504) using the manufacturer’s protocol for isolation of DNA from stool for pathogen detection. The following modifications were made: all heated incubations were at 95^o^C. For step two of the protocol, stool samples were homogenized by adding 1.4 ml ASL buffer to each sample and placing in the disruptor Genie (Scientific Industries Cat. No. SI-DD238) for 2-3 minutes or until stool was thoroughly homogenized.

**PCR amplification**

Amplification was carried out with between 2-5 ng template DNA, GoTaq hot start 2x colorless master mix from Promega (Cat. No. M5131) plus the following barcoded primers, targeting the V6 region of the 16S rRNA gene, at 0.8 pm/μL.

Forward primer: 5ACACTCTTTCCCTACACGACGCTCTTCCGATCTnnnn[BC8mer]CWACGCGARGAACCTTACC3

Reverse primer: 5CGGTCTCGGCATTCCTGCTGAACCGCTCTTCCGATCTnnnn[BC8mer]ACRACACGAGCTGACGAC3

Cycling conditions included 1 minute activation of the Taq at 95^o^C, followed by 25 cycles of 95^o^C, 55^o^C, 72^o^C at one minute each. Amplified samples were quantified using a QBit and the dsDNA reagent and pooled at equimolar amounts. Pooled samples were purified on a PCR cleanup column, and the eluted library was diluted 1:100 in deionized water and amplified using the following primers in order to attach Illumina adapters.

OLJ139:
5AATGATACGGCGACCACCGAGATCTACACTCTTTCCCTACACGA3
OLJ1403
5CAAGCAGAAGACGGCATACGAGATCGGTCTCGGCATTCCTGCTGAAC3

The PCR was as above, except with an annealing temperature of 60^o^C and 10 cycles. The product was purified and 10% phiX174 DNA was added. This library was loaded onto an Illumina MiSeq instrument with a paired-end 2x100 cycle format.

Paired end reads were assembled using XORRO^1^, and only sequences without ambiguous bases were retained for further analysis. Assembled sequences were quality-filtered and binned into OTUs with UPARSE v7.1^2^. Briefly, assembled reads were abundance-sorted and clustered into 97% similarity OTUs. Representative sequences (excluding singletons) from each OTU were further chimera-filtered against the gold database (<http://drive5.com/uchime/gold.fa>). Reads were then mapped backed to OTUs and converted into an OTU table.

**Germ-free mouse model of human fecal transplantation.**

**Mice*.*** 3-week-old germ-free male Swiss-Webster mice were purchased from Jackson laboratories. Mice were maintained in a specific-pathogen-free environment at the University of British Columbia pharmaceutical sciences animal care facility.

**Fecal transplantation.** Experimental design closely followed the rationale of previously published studies, employing germ-free models of fecal transplantation in specific pathogen free facilities^3^. Three Canadian and three South African male stool samples were selected for fecal transplantation. Each sample was used to gavage three mice. The gavage was performed immediately after the mice were removed from germ-free shipment cages, and immediately following gavage, the mice were placed in cages with filtered, sterile, viral-free air and enough food to sustain the animals over the course of the three-week experiment. This method allowed the cages to remain sealed until the experimental endpoint. Mice receiving gavage from the same samples were co-housed, as in a previously published study^4^. Stool samples were prepared by diluting 100 mg of stool into 10 mL of reduced PBS (PBS + 0.05% cysteine), in an anaerobic chamber. Mice were then gavaged with 100 uL of fecal homogenate every second day for 3 gavages starting immediately upon arrival to the animal care facility. Mice were maintained for 21 days and then sacrificed.

**Lactulose:Mannitol ratio*.*** To assess the gut barrier integrity of experimental animals, six mice per experimental group were randomly selected 24 hours prior to sacrifice. Details for this assay were previously published^5^. Briefly, all mice were gavaged with a sugar probe solution of 100 mg sucrose, 12 mg lactulose, 8 mg mannitol, and 6 mg sucralose dissolved in a 0.2 ml volume. Mice were then immediately placed in metabolic cages with no food for 22 hours. Urine was collected during this time and prepared for high-performance liquid chromatography (HPLC) according to a published protocol^6^.

**Splenocyte TLR stimulation*.*** TLR stimulation of mouse splenocytes used an adaptation of the protocol used for the human whole blood stimulation assay. 96-well plates were prepared with 22 μl of RPMI-1640 medium (Gibco Cat. No. 61870) with TLR agonists at 10-times their final concentration. The TLR agonists used were LPS (Invivogen; Cat. No. Tlrl-plps) and R848 (Invivogen Cat. No. Tlrl-r848-5), both at a final concentration of 0.1 μg/ml. At the time of sacrifice, spleens were immediately removed and stored in 5 ml sterile RPMI-1640 medium. Spleens were then disrupted using the frosted glass slide method, passed through a 70-μm filter, washed with 10 ml sterile dulbecco’s phosphate-buffered saline (dPBS; Gibco Cat. No. 14190), and centrifuged at 1400 rpm for 5 minutes at 4^o^C. Cell pellets were suspended in 4 ml red blood cell lysis buffer and incubated at room temperature for 4 minutes. 10 ml RPMI-1640 was then added to neutralize the RBL. Cells were centrifuged at 1400 rpm for 5 minutes at 4^o^C, the supernatant was discarded, and the pellet was suspended in 10 ml sterile R10 medium (RPMI-1640 supplemented with 10% fetal bovine serum). 10^6^ splenocytes in a total volume of 200 μl were added to each well containing either TLR agonists or RMPI media alone. Plates were incubated at 37^o^C for 24 hrs. Following incubation, cell cultures were centrifuged at 250 x g and 130 μl of supernatant was stored at -80^o^C until later use.

**Mouse Luminex Assay.** The eBioscience procartaplex mouse basic lumiex kit (eBioscience Cat. No. EPX010-20440-901) was used to measure cytokine concentrations in cell supernatants, including the analytes IFN-α (eBioscience Cat. No. 26027), IFN-γ (eBioscience Cat. No. 20606), IL-10 (eBioscience Cat. No. 20614), IL-23p19 (eBioscience Cat. No. 26017), IL-6 (eBioscience Cat. No. 20603), MIP-1β (eBioscience Cat. No 26014), and TNF-α (eBioscience Cat. No. 20607). Supernatants were diluted 1:1 with sterile R10 medium prior to Luminex analysis using the manufacturer’s vacuum-manifold protocol. Data were acquired on the Luminex 100 analyzer. Raw MFI data were analyzed using the MasterPlex software. 5-parameter logistic fits were used to construct standard curves for each analyte and calculate the sample concentrations. Samples with fewer than 50 beads counted were excluded from further analysis.

**16S amplicon sequencing: Fecal transplantation experiment**

**Fecal and intestinal tissue DNA extraction.** Immediately after sacrifice, the mice were sacrificed, one stool pellet and a 10 – 30 μg section of both ileum and jejunum tissue were collected from each mouse and stored in sterile 1.5 ml microcentrifuge tubes. Samples were stored at -80^o^C until DNA extraction. Total DNA was extracted from each mouse sample plus samples of the human feces used for transplant, using the Qiagen Fast DNA stool mini kit (Qiagen Cat. No. 51604) and following the protocol for stool pathogen detection with the following modifications: To disrupt the stool and intestinal samples, they were placed in a 2.0-ml Eppendorf tube with one sterile 5-mm metal bead (Qiagen Cat. No. NC9257481) and 1 ml Inhibitex buffer. Samples were then homogenized using a Disruptor Genie for 2 minutes or until samples were thoroughly homogenized. The resulting suspension was heated at 95^o^C for 5 minutes.

**PCR amplification*.*** To analyze the microbial communities in mouse tissues and feces, alongside re-extracted human stool used for the experiment, we used amplicon sequencing targeting the V4 region of the 16S rRNA gene. PCR amplification was done using 1-10 ng of DNA, Thermo Phusion Hot Start II DNA Polymerase (ThermoFisher Cat. No. F549S), and the following primers:

16Sf V4

 GTGCCAGCMGCCGCGGTAA

16Sr V4

 GGACTACHVGGGTWTCTAAT

PCR cycle conditions were 98^o^C for 2 minutes, followed by 30 cycles of 98^o^C for 20 seconds, 55^o^C for 15 seconds, and 72^o^C for 30 seconds, and 72^o^C for 10 minutes following the cycling steps. PCR products were cleaned using the Agencourt Ampure XP beads (Beckman Coulter Cat. No. A6388) using a 0.8:1 bead-sample ratio and eluted in a final volume of 20 μl.

**Library preparation*.*** Library preparation was performed using a previously published SOP^7^ with the details and product information outlined below. 10 μl of the final product was used to normalize using the SepalPrep Normalization Prep Plate Kit (ThermoFisher Cat. No. A1051001) to 1-2 ng/μl and 5 μl of each normalized sample was pooled into a single library per 96-well plate. Library pools were further concentrated using the DNA Clean and Concentrator kit (Zymo Cat. No. D4013). A dilution series was performed for each of the pooled libraries for subsequent quality control steps. Each pool was analyzed using the Agilent Bioanalyzer using the High Sensitivity DS DNA assay (Agilent Cat. No. 5047-4626) to determine approximate fragment size, and to verify library integrity. Library pools with unintended amplicons were purified using the Qiagen QIAquick Gel Extraction Kit (Qiagen Cat. No. 28706). Pooled library concentrations were determined using the KAPA Library Quantification Kit for Illumina (KAPA Cat. No KK4824). The final libraries were loaded at 8 pM, with an additional PhiX spike-in of 20%. The amplicon library was sequenced on the MiSeq using the MiSeq 500 Cycle V2 Reagent Kit (Illumina Cat. No. MS-102-2003).

**Read processing and OTU binning.** Paired-end reads were assembled using MOTHUR version 1.37.2, following the MiSeq SOP^7^ [accessed on Apr 2107]. OTUs were clustered at 97% identity and classified using the GreenGenes 13_8_99 database^8^.

**Statistical Analysis.**

**Data preparation**

**Cytokine data preparation for multivariate analysis.** Because baseline, or unstimulated data cannot be incorporated into sPLS-DA, we selected only cytokines whose variance significantly differed from unstimulated control values using the Fligner-Killeen test. For baseline values, we selected only cytokines where at least 70% of subjects had unstimulated values above the detection limit, with a median fluorescence intensity (MFI) greater than 10. The resulting cytokine-stimulus combinations were used to create a matrix of concentration values. The data were log10-normalized. Subjects with missing values for 15% or more features and cytokines with missing values for 15% or more subjects were removed. The remaining missing values were imputed using the *missForest* R package^9^. Cytokine responses to PRR agonists that characterized each cohort in a multivariate space were selected using sPLS-DA.

**OTU pre-filtering for integration.**  Following analysis of alpha and beta-diversity, data were further filtered to remove rare OTUs using two criteria. First, we removed all near zero variance OTUs using the nearZeroVar function in mixOmics^10^ with the frequency cutoff set to 90/10 and unique cutoff set to 10. Next, we removed rare OTUs by retaining only OTUs that had counts above 3 in at least 5% of samples. After filtering, 1076 OTUs were retained for subsequent analyses.

**OTU data normalization.** The OTU data was first total sum of squares (TSS) transformed. The centered-log-ratio (CLR) transformation was then applied to normalize OTU data. Because CLR is a log-transformation, it cannot accommodate zeroes. Thus, an offset to all zero values was added as 0.1 times the minimum relative abundance in each sample (the default offset applied in *mixOmics* version 6). To better suit the regression framework of sPLS, we limited our analysis to OTUs present in at least 50% of the samples included in each analysis.

**Microbiome analysis**

**Alpha diversity.** We estimated abundance-dependent microbial diversity with the Shannon diversity index or abundance-independent diversity by calculating the observed richness, after subsampling the OTU table to 30,246 reads per sample to normalize sampling depth. To test whether either richness or diversity differed among the four cohorts, we applied the Kruskal-Wallis test, followed by the Dunn’s post-test, where significant relationships were found. P-values were adjusted using the Bonferroni method. To test for relationships between host factors and Shannon diversity, we used linear regression, allowing assessment of multiple host factors within the same model. To test for a quadratic relationship between Shannon diversity and maternal age in the combined Ecuadorean and Canadian cohorts, we fitted a linear model with B-spline transformed maternal age, using 5 knots and setting degrees to two (quadratic relationship) using the R package *splines* implemented through base R.

**Community composition**. We used the *phyloseq* R package^11^ to compute alpha and beta diversity. To identify subject characteristics potentially explaining multivariate community composition, we used the forward model selection algorithm implemented in the R package *vegan* (function *ordiR2step*)^12^. This procedure minimizes Type I errors and corrects for the overestimation of the proportion of explained variance by selected variables. The significance (α < 0.05) of variables was evaluated stepwise and the order of variable evaluation was based on improvement in the model’s adjusted R^2^. Model selection proceeded until the next independent variable was non-significant as determined by 1,000 permutations. Missing values in the metadata were imputed via random forest analysis using the *missForest* R package^9^.

**Differential Abundance.** To identify OTUs whose normalized abundance significantly differed between cohorts, we used the likelihood ratio test implemented in the R DESeq2 package^13^.

**Identifying cohort-specific OTUs.** To further characterize OTUs that classified each cohort in a multivariate space, we used sparse Partial Least Squares Discriminant Analysis (sPLS-DA)^14^ of variance-stabilized counts of the differentially abundant OTUs. Differentially abundant OTUs were graphically represented by heatmaps using the *pheatmap* R package^15^.

**Integrated analysis of cytokine and microbiome data**

**sPLS Integration**

The number of features selected per component was set to a maximum of the number of samples in the dataset, and further reduced by limiting the selection to features that significantly correlated using the Pearson correlation to at least 30% of the features in the other data type. Cytokine-OTU relationships were displayed using heatmaps generated with the *pheatmap* R package. Networks were generated using the *igraph* R package^16^ and exported as *graphml* objects for further editing in Cytoscape^17^.

**sPLS Integration of host demographic, immune, and microbiome data.** To determine whether any stool microbiome-systemic innate immune response correlations also covaried with demographic data, we performed a block-sPLS analysis, whereby each of the three data sets (microbiome, innate immune, and host demographic) constitute individual blocks, with feature selection constrained to co-vary with each data type. Breast feeding was incorporated in this analysis both as time since breast feeding (time difference between cessation of breast feeding and age at time of sampling) and duration of breast feeding. Other host factors incorporated into this analysis were delivery mode, child sex, WAZ, HAZ, WLZ, maternal age, and gestational age. This analysis was performed for Canadian and Ecuadorean children, where adequate sample size and group representation reduced the chance of identifying spurious correlations. Models were built for Canadian and Ecuadorean children together, and for each cohort separately.

**Over-representation analysis.** We used the *phyper* function in base R to test for stimulus, cytokine, and bacterial family over-representation within each sPLS analysis. The analysis was done for each sPLS component separately, and only features that were present four or more times in the input data were included in the test. This was done because the selection of singleton feature classes, especially relevant for OTU data, results in spurious identification of enriched classes. P-values were adjusted for each component separately using the *Bonferroni* correction. All significantly over-represented classes had an adjusted p-value under 0.05.

**References**

1. J. Dickson R, Gloor G. XORRO: Rapid Paired-End Read Overlapper; 2013.

2. Edgar RC. UPARSE: highly accurate OTU sequences from microbial amplicon reads. Nat Methods 2013; 10:996-8.

3. Arrieta MC, Stiemsma LT, Dimitriu PA, Thorson L, Russell S, Yurist-Doutsch S, Kuzeljevic B, Gold MJ, Britton HM, Lefebvre DL, Subbarao P, Mandhane P, Becker A, McNagny KM, Sears MR, Kollmann T, Mohn WW, Turvey SE, Finlay BB. Early infancy microbial and metabolic alterations affect risk of childhood asthma. Sci Transl Med 2015; 7:307ra152.

4. Blanton LV, Charbonneau MR, Salih T, Barratt MJ, Venkatesh S, Ilkaveya O, Subramanian S, Manary MJ, Trehan I, Jorgensen JM, Fan YM, Henrissat B, Leyn SA, Rodionov DA, Osterman AL, Maleta KM, Newgard CB, Ashorn P, Dewey KG, Gordon JI. Gut bacteria that prevent growth impairments transmitted by microbiota from malnourished children. Science 2016; 351.

5. Arrieta MC, Madsen K, Doyle J, Meddings J. Reducing small intestinal permeability attenuates colitis in the IL10 gene-deficient mouse. Gut 2008; 58:41-8.

6. Meddings JB, Gibbons I. Discrimination of site-specific alterations in gastrointestinal permeability in the rat. Gastroenterology 1998; 114:83-92.

7. Kozich JJ, Westcott SL, Baxter NT, Highlander SK, Schloss PD. Development of a dual-index sequencing strategy and curation pipeline for analyzing amplicon sequence data on the MiSeq Illumina sequencing platform. Appl Environ Microbiol 2013; 79:5112-20.

8. DeSantis TZ, Hugenholtz P, Larsen N, Rojas M, Brodie EL, Keller K, Huber T, Dalevi D, Hu P, Andersen GL. Greengenes, a Chimera-Checked 16S rRNA Gene Database and Workbench Compatible with ARB. Applied and Environmental Microbiology 2006; 72:5069-72.

9. Stekhoven DJ, Buhlmann P. MissForest--non-parametric missing value imputation for mixed-type data. Bioinformatics 2012; 28:112-8.

10. Rohart F, Gautier B, Singh A, Lê Cao K-A. mixOmics: An R package for ‘omics feature selection and multiple data integration. PLOS Computational Biology 2017; 13:e1005752.

11. Charlop-Powers Z, Brady SF. phylogeo: an R package for geographic analysis and visualization of microbiome data. Bioinformatics 2015; 31:2909-11.

12. Oksanen J, Blanchet G, Friendly M, Kindt R, Legendre P, McGlinn D, Minchin PR, O'Hara RB, Simpson GL, Solymos P, Stevens MHH, Szoecs E, Wagner H. vegan: Community Ecology Package. 2017. <https://CRAN.R-project.org/package=vegan>,

13. Love MI, Huber W, Anders S. Moderated estimation of fold change and dispersion for RNA-seq data with DESeq2. Genome Biol 2014; 15:550.

14. Le Cao KA, Boitard S, Besse P. Sparse PLS discriminant analysis: biologically relevant feature selection and graphical displays for multiclass problems. BMC Bioinformatics 2011; 12:253.

15. Kolde R. pheatmap: Pretty Heatmaps. 2015. <https://CRAN.R-project.org/package=pheatmap>,

16. Csardi G, Nepusz T. The igraph software package for complex network research. InterJournal 2006; Complex Systems:1695.

17. Shannon P, Markiel A, Ozier O, Baliga NS, Wang JT, Ramage D, Amin N, Schwikowski B, Ideker T. Cytoscape: a software environment for integrated models of biomolecular interaction networks. Genome Res 2003; 13:2498-504.
